# Activated Platelet–Released Heat Shock Protein 90α Triggers Autophagy‐Dependent Neutrophil Extracellular Trap Formation and Amplifies Sepsis

**DOI:** 10.1002/advs.202515933

**Published:** 2026-02-24

**Authors:** Chengbo Wang, Maodong Leng, Chenchen Sun, Jingyu Cao, Linfei Li, Yangyang Jia, Yongshuai Han, Yuchun Liu, Yaodong Zhang, Chenglong Zhang, Yingli Men, Ningyuan Liu, Yibing Cheng, Yixia Zhang, Ya Li, Zhenlong Li, Lidan Cui, Xiangzhan Zhu

**Affiliations:** ^1^ Henan Clinical Research Center of Childhood Diseases Henan Key Laboratory of Children's Genetics and Metabolic Diseases Henan Children's Hospital Zhengzhou Children's Hospital Children's Hospital Affiliated to Zhengzhou University Zhengzhou China; ^2^ Reproductive Medicine Center The Third Affiliated Hospital of Zhengzhou University Zhengzhou China; ^3^ Department of Head and Neck Surgery Anyang Tumor Hospital Affiliated to Henan University of Science and Technology Anyang Tumor Hospital Anyang Henan China; ^4^ School of Life Science Henan Institute of Science and Technology Xinxiang China; ^5^ Translational Medicine Research Center Zhengzhou People's Hospital The Fifth Clinical College of Henan University of Chinese Medicine Zhengzhou China; ^6^ Department of Radiology The Second Xiangya Hospital Central South University Changsha China; ^7^ Zhengzhou Children's Hospital PICU Henan Children's Hospital, Zhengzhou Children's Hospital Children's Hospital Affiliated to Zhengzhou University Zhengzhou China; ^8^ Henan Key Laboratory of Helicobacter Pylori Microbiota and Gastrointestinal Cancer Marshall Medical Research Center The Fifth Affiliated Hospital of Zhengzhou University Zhengzhou China; ^9^ Department of Hematology and Hematopoietic Cell Transplantation City of Hope National Medical Center Los Angeles California USA; ^10^ School of Life Sciences Zhengzhou University Zhengzhou China

**Keywords:** platelet, HSP90α, NET, autophagy, sepsis

## Abstract

Platelets are crucial to the development of thrombosis and coagulation abnormalities in sepsis, but the mechanisms by which they contribute to these pathological processes are not fully understood. Here, we identify a key role for platelet‐released heat shock protein 90α (HSP90α) in driving neutrophil extracellular trap (NET) formation and supporting thromboinflammation during sepsis. Proteomic analysis of platelets from patients with sepsis showed a significant increase in HSP90α, which we traced back to trafficking pathways originating from megakaryocytes. When activated, platelets translocate HSP90α to their plasma membrane and release it into the extracellular space in both free and exosome‐associated forms. Extracellular HSP90α acts as a damage‐associated molecular pattern that binds to toll‐like receptor 4 (TLR4) on neutrophils. This binding activates a downstream MyD88–Beclin 1 signaling pathway, triggering autophagy and leading to NET formation. Blocking extracellular HSP90α with a neutralizing monoclonal antibody significantly reduced NET formation both in vitro and in vivo, resulting in decreased sepsis‐related thrombosis and inflammation. This platelet–HSP90α–TLR4–autophagy–NET pathway not only deepens our understanding of platelet‐induced immunothrombosis but also suggests potential targets for therapies aimed at reducing coagulation problems and organ failure in septic patients.

## Graphical abstract

HSP90α is significantly upregulated in platelets from sepsis patients, with its origin from megakaryocyte‐derived trafficking. Furthermore, activated platelets secrete HSP90α into the extracellular space in both free and exosome‐associated forms. Finally, extracellular HSP90α directly engages TLR4 on neutrophils to induce autophagy, leading to NET formation—especially via the MyD88–Beclin 1 pathway. This specific platelet–eHSP90α–TLR4–autophagy–NET axis reveals a novel mechanism linking platelet activation to thromboinflammation and highlights a potential therapeutic target for managing coagulation disorders in sepsis.

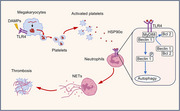

## 1 Introduction

Sepsis is a life‐threatening condition characterized by organ dysfunction resulting from a dysregulated host response to infection [1]. This overwhelming inflammatory response can lead to widespread organ impairment, including coagulopathy such as disseminated intravascular coagulation (DIC) [2]. Coagulopathy is a key contributor to multi‐organ failure and increased mortality in sepsis [3]. Therefore, a deeper understanding of the mechanisms driving sepsis‐induced coagulopathy is critical for developing effective therapeutic strategies.

Platelets, derived from megakaryocytes during maturation, are traditionally recognized for their essential role in homeostasis [4]. Beyond this classical function, platelets also contribute to immune responses during inflammation [5]. Upon activation, platelets can promote neutrophil extracellular trap (NET) formation through direct interactions with neutrophils or via the release of mediators such as von Willebrand factor (vWF), platelet factor 4 (PF4), and thromboxane A2 (TXA2) [6]. In turn, components of NET (e.g., histones and neutrophil elastase) enhance platelet aggregation, fibrin formation, and thrombus development [7]. In sepsis, both platelet activation and NET formation are markedly elevated and contribute to DIC and multiorgan dysfunction [8]. However, the molecular mechanisms by which platelets initiate or amplify NET formation under septic conditions remain poorly understood. Uncovering these pathways is critical, as targeting the platelet–NET axis may offer novel therapeutic opportunities to mitigate thromboinflammation and organ failure in sepsis.

Heat shock protein 90 (HSP90) is a family of ATP‐dependent molecular chaperones that regulate the folding, maturation, and post‐translational modification of a wide array of client proteins [9]. Two major isoforms exist: the stress‐inducible HSP90α and the constitutively expressed HSP90β [10]. HSP90α can be actively secreted into the extracellular space, where it functions as a danger‐associated molecular pattern (DAMP) to activate components of the innate immune system [11]. While elevated levels of extracellular HSP90α (eHSP90α) have been reported in the serum of patients with sepsis and are associated with disease severity and multi‐organ dysfunction [12], the presence and function of it in sepsis remain undefined.

Here, we demonstrated that HSP90α expression is upregulated in platelets from patients with sepsis and that it is mobilized to the extracellular space upon platelet activation. eHSP90α was found to trigger NET formation by inducing autophagy through the TLR4/MyD88/Beclin 1 signaling pathway. Blockade of eHSP90α significantly reduced NET‐driven thrombosis. These findings identify eHSP90α as a key mediator of platelet–NET interactions and highlight it as a potential therapeutic target for sepsis‐associated thrombosis.

## 2 Results

### 2.1 Platelets from Patients with Sepsis Exhibited Elevated Levels of HSP90α

**FIGURE 1:**
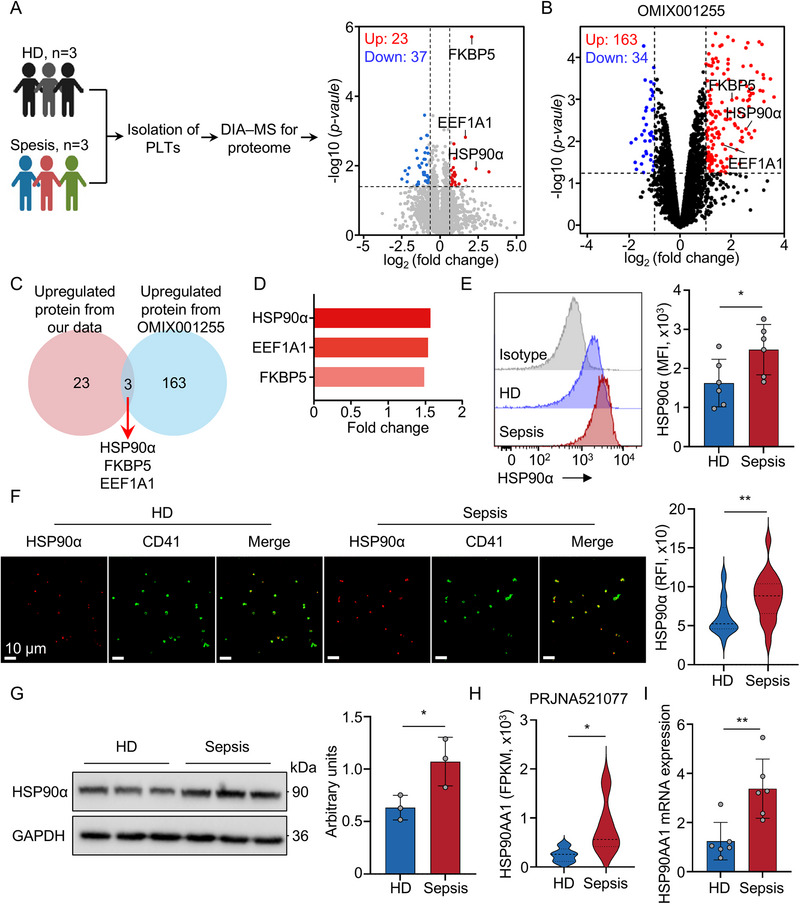
The platelet proteomic analysis reveals increased HSP90α expression in patients with sepsis. (A) Schematic diagram of the experimental design for DIA‐MS (HD, *n* = 3; sepsis, *n* = 3). Volcano plot with significantly increased (red) and decreased (blue) expression of proteins from the HD and sepsis groups. Cutoff: fold change > 1.5 and P value < 0.05. (B) Volcano plot showing significantly increased (red) and decreased (blue) expression of proteins in the HD and severe sepsis groups from OMIX001255. Cutoff: fold change > 2 and P value < 0.05. (C) Venn diagram showing overlap in increased protein expression between our data and OMIX001255. (D) The fold change of HSP90α, FKBP5 and EEF1A1 in HD and sepsis groups. (E) Representative flow cytometry histograms and quantification for HSP90α in platelets from HD (*n* = 6) and sepsis patients (*n* = 6). (F) Representative immunofluorescence microscopy of HSP90α (red) and CD41 (green) was performed in platelets from HD and sepsis patients. Scale bar: 10 µm. Relative HSP90α fluorescence intensity was quantified by Image J. (G) Immunoblot and quantification analysis for HSP90α in platelets from HD (*n* = 3) and sepsis patients (*n* = 3). (H) Violin plots with *HSP90AA1* expression (FPKM) in platelets of HD and sepsis patients from the publicly RNA‐seq data (PRJNA521077). (I) HSP90α mRNA was quantified in platelets from HD (*n* = 6) and sepsis patients (*n* = 6). All data are presented as the mean ± SD. Statistical analysis was conducted using unpaired two‐tailed *t*‐test (E–G, I). ^*^ *P* < 0.05, ^**^ *P* < 0.01. Each data point represents one human platelet sample (E, F, G, I).

A retrospective review of electronic medical records of patients with sepsis (*n* = 20) and healthy donors (HD; *n* = 20) showed that patients with sepsis exhibited signs of coagulopathy, including elevated activated partial thromboplastin time (aPTT) and prolonged prothrombin time (PT) (Table S1). Considering the important role of platelets in coagulation [13], we next performed high‐throughput proteomic analysis using data‐independent acquisition mass spectrometry (DIA‐MS) on purified platelets from three patients with sepsis and three HD (Figure 1A). A total of 4627 proteins were identified, with 4325 quantified across all samples. Compared to platelets from HD, platelets from patients with sepsis exhibited 23 upregulated and 37 downregulated proteins (Figure 1A; Figure S1A). Reactome pathway analysis of the 23 upregulated proteins revealed significant enrichment in pathways related to autophagy and the cellular response to heat stress in platelets from patients with sepsis (Figure S1B,C). We also analyzed a publicly available platelet proteomics dataset (OMIX001255), identifying 163 up‐regulated and 34 down‐regulated proteins in platelets from patients with severe sepsis (Figure 1B; Figure S1D). Gene ontology (GO) analysis of the 163 upregulated proteins revealed significant enrichment in pathways related to platelet adhesion and granule release (Figure S1E). Cross‐comparison between the two datasets identified three upregulated proteins: HSP90α, FKBP5, and EEF1A1 (Figure 1C). Among them, HSP90α exhibited the highest fold change between samples from HD and patients with sepsis (Figure 1D). Elevated HSP90α levels in septic platelets were validated by flow cytometry, immunofluorescence, and western blotting (WB) (Figure 1E–G). In addition, the increased FKBP5 and EEF1A1 mRNA expression in septic platelets was validated by quantitative real‐time polymerase chain reaction (qRT‐PCR) (Figure S1F,G). Consistent with our findings, FKBP5 has been shown to regulate platelet activation and thrombosis [14, 15]. In addition, platelet RNA sequencing from patients with Parkinson's disease—who exhibit platelet dysfunction—revealed significantly elevated EEF1A1 expression, identifying it as a potential biomarker [16]. Taken together, these results indicate that platelet activation in sepsis is characterized by upregulation of HSP90α.

### 2.2 Megakaryocyte HSP90α is Upregulated via Toll‐Like Receptor 4 Signaling and then Delivered to Platelets

Transcriptomic analysis of platelet bulk RNA‐seq data (PRJNA521077 [17]) demonstrated elevated *HSP90AA1* expression, the gene encoding HSP90α, in platelets from patients with sepsis relative to HD (Figure 1H; Figure S2A), a finding further validated by qRT‐PCR (Figure 1I). Given that platelets are anucleate and incapable of de novo transcription, and that their RNA is inherited from megakaryocytes [18], the elevated levels of HSP90α mRNA observed in sepsis platelets likely originate from increased transcriptional activity in megakaryocytes and subsequent RNA transfer. Supporting this, analysis of both megakaryocytes and platelets from two mouse models of sepsis—cecal ligation and puncture (CLP) and lipopolysaccharide (LPS) challenge—revealed that significantly elevated *Hsp90aa1* expression in both cell types compared to sham‐treated controls (Figure S2B–E). These findings support the conclusion that megakaryocytes are the source of increased platelet HSP90α during sepsis. Toll‐like receptor 4 (TLR4) has been shown to enable megakaryocytes to sense infection and inflammatory signals [19]. To determine whether the upregulation of HSP90α in megakaryocytes during sepsis is TLR4‐dependent, we examined *Hsp90aa1* expression in TLR4^−/−^ septic mice. Compared to wild‐type (WT) controls, *Hsp90aa1* expression was significantly reduced in megakaryocytes from TLR4^−/−^ septic mice, and this reduction was similarly reflected in platelets (Figure S2F–I). To confirm the pivotal role of TLR4 in mediating the HSP90α mRNA expression, the megakaryocytes of WT and TLR4^−/−^ mice were subjected to treatment with platelet‐poor plasma (PPP) from WT CLP mice or WT sham mice. As expected, TLR4 deficiency abrogated the effect of CLP PPP on increased HSP90α mRNA expression in megakaryocytes (Figure S2J). Furthermore, TLR4 deficiency also abolished the elevated phosphorylated nuclear factor κB (p‐NFκB) levels in megakaryocytes after CLP (Figure S2K). These findings suggest that TLR4 signaling is essential for the upregulation of HSP90α in megakaryocytes during sepsis and that the elevated levels of HSP90α in platelets originate from megakaryocyte‐derived transcripts.

### 2.3 Platelet Activation Triggers HSP90α Membrane Translocation and Release

**FIGURE 2:**
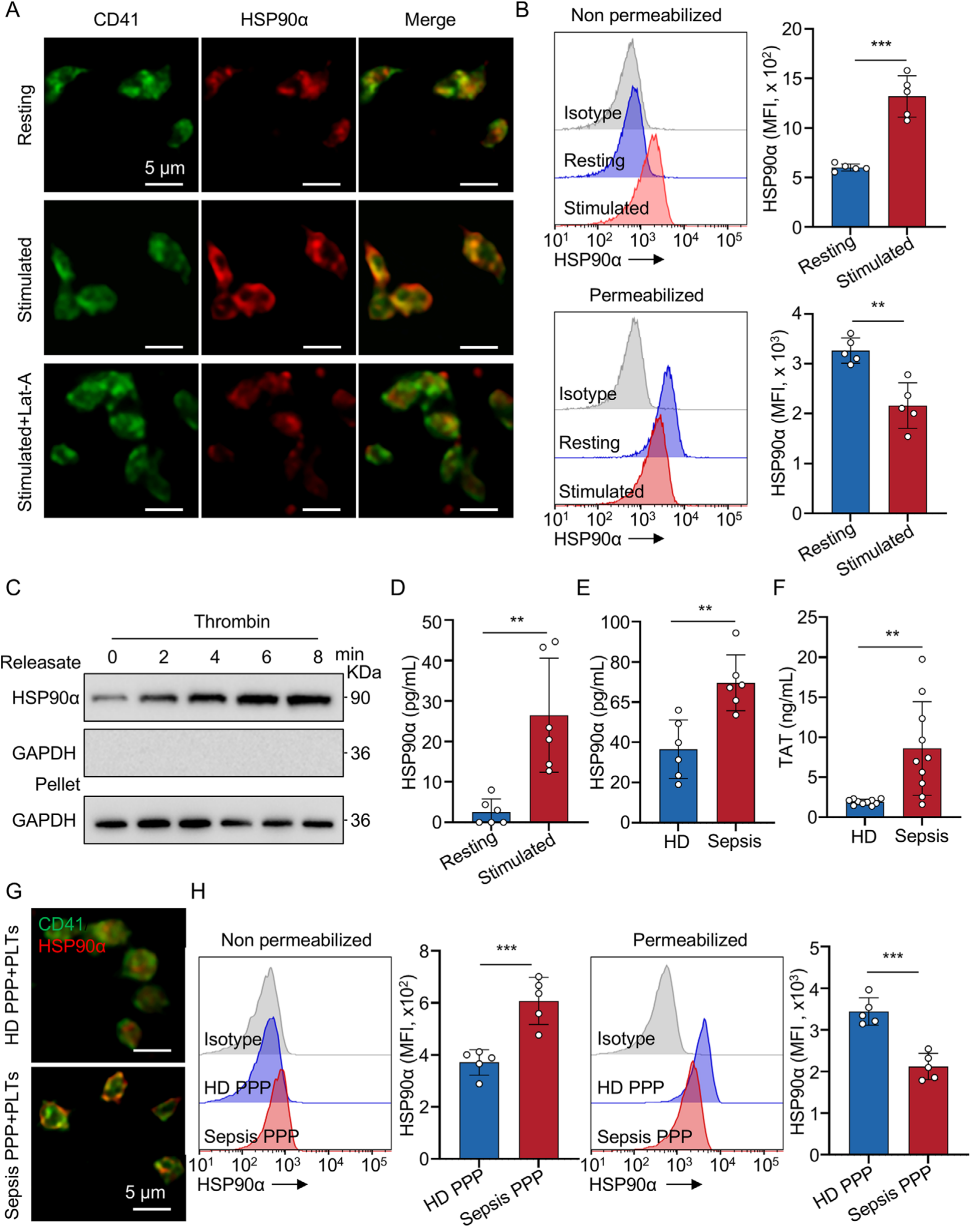
Platelet HSP90α is translocated and released following thrombin stimulation. (A) Representative immunofluorescence microscopy of HSP90α (red) and CD41 (green) was performed on HD platelets treated for 10 min with HEPES, thrombin (0.05 U/mL), and a combination of latrunculin A (200 µm) and thrombin (0.05 U/mL). Scale bars: 5 µm. (B) Representative flow cytometry histograms and quantification for HSP90α on the membrane and intracellular in resting HD platelets (*n* = 5) or HD platelets stimulated with thrombin (0.05 U/mL) for 10 minutes (*n* = 5). (C) Immunoblotting analysis of releasate and pellet in HD platelets treated with thrombin (0.05 U/ml). (D) ELISA analysis of HSP90α in the supernatant of HD platelets treated or untreated with thrombin (0.05 U/mL) (*n* = 6). (E) ELISA analysis of HSP90α in the supernatant of HD or sepsis platelets treated with thrombin (0.05 U/mL) (*n* = 6). (F) Boxplots showing quantification of TAT in the plasma of HD (*n* = 10) and sepsis patients (*n* = 10). (G) Representative immunofluorescence microscopy of HSP90α (red) and CD41 (green) was performed in platelets treated with HD and sepsis PPP. (H) Representative flow cytometry histograms and quantification for HSP90α on the membrane and intracellular in platelets treated with HD (*n* = 5) and sepsis PPP (*n* = 5). All data are presented as the mean ± SD. Statistical analysis was conducted using an unpaired two‐tailed *t*‐test (B, D, E, F, H). ^**^ *P* < 0.01, ^***^ *P* < 0.001. Each data point represents one experiment (B, H) or one human plasma sample (D, E, F). For A, B, C, G, H, three independent experiments were performed. PPP, platelet‐poor plasma; PLTs, platelets.

In resting platelets, immunofluorescence staining showed that HSP90α was diffusely distributed within the cytoplasm (Figure 2A). Upon activation by thrombin, HSP90α relocated to the plasma membrane, forming a characteristic ring‐like pattern (Figure 2A; Figure S3A). This translocation was impaired by latrunculin A (Lat‐A), an inhibitor of actin polymerization, resulting in a distribution pattern similar to that of unstimulated platelets (Figure 2A; Figure S3A). These results indicate that actin polymerization is essential for the membrane translocation of HSP90α during platelet activation. Flow cytometric analysis of resting and activated platelets further demonstrated that HSP90α expression increased at the plasma membrane and decreased intracellularly upon platelet activation (Figure 2B). In addition, compared with resting platelets, the expression level of HSP90α on the platelet membrane surface remained significantly elevated following prolonged thrombin stimulation (Figure S3B). Furthermore, immunoblotting revealed the presence of HSP90α in the extracellular fraction of platelet releasates after stimulation (Figure 2C), and enzyme‐linked immunosorbent assay (ELISA) detected the presence of HSP90α in the supernatant of thrombin‐stimulated platelets (Figure 2D), indicating that HSP90α is actively released from platelets upon activation. To determine whether the released HSP90α is free or exosome‐associated, the platelet‐derived exosomes were isolated following thrombin stimulation. Consistent with previous findings that tumor cells secrete HSP90 via exosomes, the presence of HSP90α was detected in thrombin‐stimulated platelet‐derived exosomes samples (Figure S3C,D). More importantly, even when exosome release was inhibited with GE4869, activated platelets still produced substantial amounts of HSP90α compared to resting platelets (Figure S3E). The results indicated that HSP90α, when released by platelets, exists in both free and vesicle‐associated forms.

In addition, compared with HD, the level of platelet‐derived HSP90α was significantly increased in platelets from sepsis (Figure 2E). Consistent with these findings, PPP from patients with sepsis—characterized by elevated levels of thrombin–antithrombin (TAT) complexes (Figure 2F), indicative of increased thrombin production, also could stimulate platelets to mobilize HSP90α to the membrane surface (Figure 2G). Flow cytometry further validated this effect, showing increased surface and decreased intracellular HSP90α expression following exposure to sepsis PPP (Figure 2H). Collectively, these results demonstrate that platelet activation drives HSP90α mobilization to the extracellular space via both free and exosome‐dependent pathways, and that soluble factors present in sepsis plasma are sufficient to initiate this process.

### 2.4 HSP90α Released by Platelets Enhances Sepsis‐induced Thrombosis via Neutrophils

**FIGURE 3:**
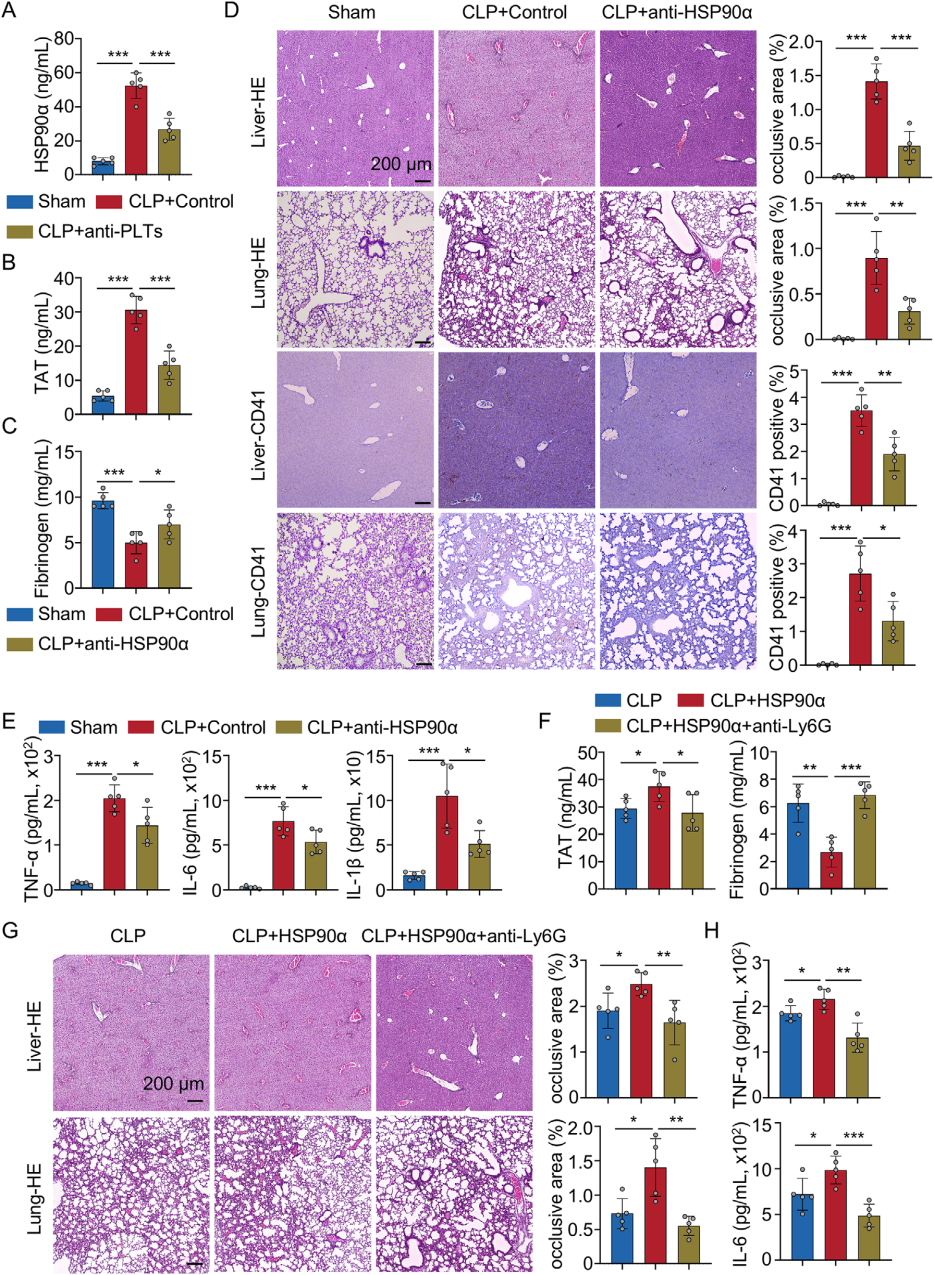
eHSP90α facilitated sepsis‐induced thrombosis by neutrophils. (A) Plasma HSP90α levels of sham and CLP‐operated mice in the absence or presence of anti‐PLTs treatment (*n* = 5). (B) Plasma TAT levels of sham and CLP‐operated mice in the absence or presence of anti‐HSP90α treatment (*n* = 5). (C) Plasma fibrinogen levels of sham and CLP‐operated mice in the absence or presence of anti‐HSP90α treatment (*n* = 5). (D) HE staining and IHC staining of CD41 in livers and lungs of sham and CLP‐operated mice in the absence or presence of anti‐HSP90α treatment (*n* = 5). HE staining statistical analysis for thrombus area in mice livers and lungs was shown (*n* = 5). IHC staining statistical analysis for platelets in mice livers and lungs is shown (*n* = 5). Scale bars: 200 µm. (E) Bar graphs displaying the levels of TNF‐α, IL‐6, and IL‐1β in plasma from each group. (F) Plasma TAT and fibrinogen levels of CLP‐operated mice, CLP‐operated mice treated with recombinant HSP90α and/or anti‐Ly6G antibody (*n* = 5). (G) HE staining in livers and lungs of CLP‐operated mice, CLP‐operated mice treated with recombinant HSP90α and/or anti‐Ly6G antibody (*n* = 5). HE staining statistical analysis for thrombus area in mice livers and lungs was shown (*n* = 5). (H) Bar graphs displaying the levels of TNF‐α and IL‐6 in plasma from each group. All data are presented as the mean ± SD. Statistical analysis was conducted using one‐way ANOVA and Holm‐Šídák's multiple comparisons test (A–H). ^*^ *P* < 0.05, ^**^ *P* < 0.01, ^***^ *P* < 0.001. Each data point represents one mouse (A–H). For A–H, three independent experiments were performed.

Consistent with observations in patients with sepsis, serum levels of eHSP90α were significantly elevated in CLP mice. Plasma HSP90α levels in platelet‐depleted mice using an anti‐platelet antibody were reduced by 49.22% ± 11.86 compared to the CLP mice treated with an isotype control antibody (Figure 3A). These results suggest that platelets are a major but not the sole source of circulating eHSP90α during sepsis. To investigate the functional role of eHSP90α in sepsis, CLP mice were treated with the HSP90α neutralizing antibody 1G6‐D7, which was developed to target a specific region that distinguished HSP90α from HSP90β [20]. Compared to the CLP mice treated with an isotype control antibody, treatment with 1G6‐D7 significantly decreased levels of TAT complexes and restored fibrinogen levels (Figure 3B,C), two key markers of disseminated intravascular DIC. Histological examination of liver and lung tissues further revealed a marked reduction in thrombus formation and platelet aggregation following 1G6‐D7 treatment, compared to the CLP mice treated with an isotype control antibody (Figure 3D). Systemic levels of pro‐inflammatory cytokines—including TNF‐α, IL‐6, and IL‐1β—were also significantly reduced in 1G6‐D7–treated mice, compared to the CLP mice treated with an isotype control antibody (Figure 3E). Moreover, the safety profile of 1G6‐D7 showed that its administration did not induce measurable hematologic, inflammatory, or organ toxicity, further highlighting the potential implications for clinical treatment (Figure S4A–E). Together, these results suggest that eHSP90α plays a critical role in promoting thromboinflammation, and that its neutralization reduces coagulation markers and thrombus formation while also dampening systemic inflammatory responses.

To investigate the mechanism underlying the pro‐thrombotic role of eHSP90α in sepsis, we analyzed a publicly available single‐cell RNA sequencing (scRNA‐seq) dataset (GSE167363 [21]). derived from peripheral blood mononuclear cells (PBMCs) of patients with sepsis. Unsupervised clustering identified 12 distinct cell clusters corresponding to major immune cell types (Figure S5A–C). Among these, neutrophils and platelets emerged as the most abundant populations (Figure S5D,E). Notably, *HSP90AA1* was the most highly expressed member of the HSP family in platelets (Figure S5F,G), consistent with our proteomic analysis. Given their established role in immunothrombosis and their dominance in the scRNA‐seq dataset, neutrophils were prioritized for further analysis as potential effectors of eHSP90α‐mediated thromboinflammatory responses in sepsis. We, therefore, administered an anti‐Ly6G antibody to selectively deplete neutrophils in septic mice. This intervention significantly reduced HSP90α‐associated TAT complexes and restored fibrinogen levels (Figure 3F), compared to the CLP mice treated with recombinant HSP90α alone. Histological analysis revealed that neutrophil depletion also significantly attenuated thrombus formation in the liver and lungs (Figure 3G). In parallel, systemic inflammation was suppressed, as evidenced by significantly decreased levels of the pro‐inflammatory cytokines TNF‐α and IL‐6 (Figure 3H), compared to the CLP mice treated with recombinant HSP90α alone. Similar results were found in LPS‐treated mice (Figure S6A–H). These results suggest that neutrophils are critical contributors to the thromboinflammatory response triggered by eHSP90α during sepsis.

### 2.5 eHSP90α Triggers Neutrophil Extracellular Trap Formation by Activating Autophagy in Neutrophils

**FIGURE 4:**
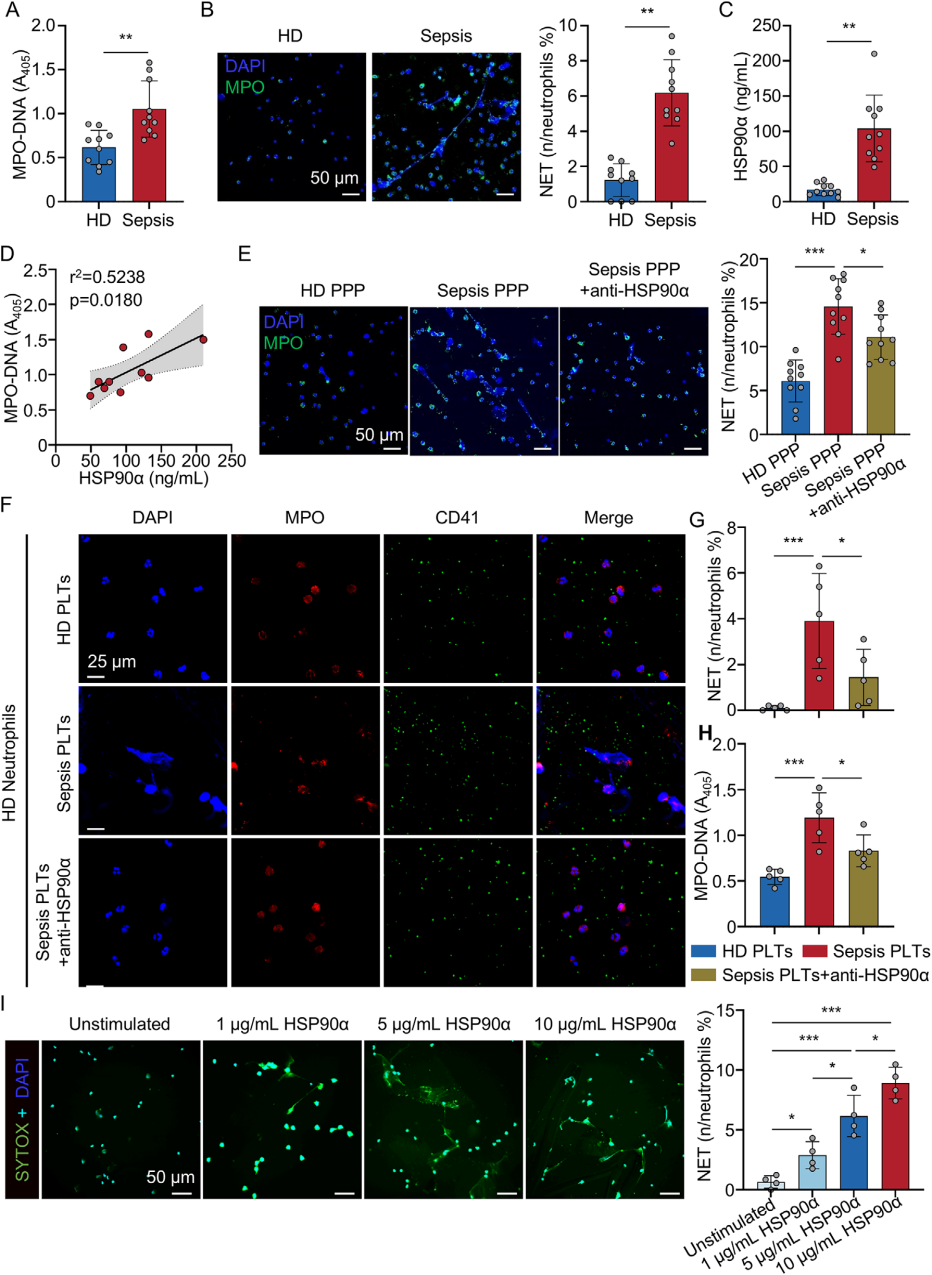
Sepsis platelets promote NET formation via eHSP90α. (A) NET markers MPO‐DNA were measured in plasma samples from HD (*n* = 10) and sepsis patients (*n* = 10) by ELISA. (B) Immunofluorescence and quantification analysis of NETs in neutrophils from HD (*n* = 10) and sepsis patients (*n* = 10). Scale bars: 50 µm. Cells were stained with DAPI for DNA (blue) and anti‐MPO for PMNs or NETs (green). (C) Boxplots showing quantification of HSP90α in the plasma of HD (*n* = 10) and sepsis patients (*n* = 10). (D) Evaluate the correlation between serum HSP90α and MPO‐DNA concentration in sepsis patients (*n* = 10). (E) Immunofluorescence and quantification analysis of NETs in neutrophils treated with HD PPP (*n* = 10), sepsis PPP (*n* = 10) and anti‐HSP90α preincubated sepsis PPP (*n* = 10). Cells were stained with DAPI for DNA (blue) and anti‐MPO for PMNs or NETs (green). Scale bars: 50 µm. (F,G) Immunofluorescence and quantification analysis of NETs following in vitro co‐culture of neutrophils with HD PLTs (*n* = 5), sepsis PLTs (*n* = 5) and anti‐HSP90α preincubated sepsis PLTs (*n* = 5). Scale bars: 25 µm. (H) Quantification of MPO‐DNA in the supernatant of neutrophils from each group (*n* = 5). (I) Immunofluorescence and quantification analysis of NET‐forming neutrophils following recombinant HSP90α stimulation (*n* = 4). Scale bars: 50 µm. All data are presented as the mean ± SD. Statistical analysis was conducted using an unpaired two‐tailed t‐test (A–C), one‐way ANOVA and Holm‐Šídák's multiple comparisons test (E–I) and two‐tailed Pearson's correlation test (D) ^*^ *P* < 0.05, ^**^ *P* < 0.01, ^***^ *P* < 0.001. Each data point represents one human sample (A–D) or one experiment well (E–I). For E–I, three independent experiments were performed. PPP, platelet‐poor plasma; PLTs, platelets.

Analysis of myeloperoxidase (MPO)‐DNA complexes, compared to HD, we found that neutrophil extracellular trap (NET) formation was significantly increased in the plasma of patients with sepsis (Figure 4A). Consistently, the frequency of NET‐releasing neutrophils was significantly elevated in patients with sepsis (Figure 4B). We also found that plasma HSP90α levels were significantly increased and positively correlated with NET formation in patients with sepsis (Figure 4C,D), suggesting a potential role for HSP90α in this process.

To directly test the function of eHSP90α, we incubated purified neutrophils with sepsis‐derived PPP in the presence or absence of the neutralizing antibody 1G6‐D7. Blockade of eHSP90α using a neutralizing antibody significantly reduced PPP‐induced NET formation (Figure 4E), compared to treatment without the antibody. Similarly, treatment with 1G6‐D7 significantly suppressed NET formation induced by sepsis‐derived platelets, as evidenced by a reduction in MPO–DNA complexes (Figure 4F–H), compared to treatment without 1G6‐D7. Moreover, stimulation of neutrophils with recombinant HSP90α enhanced NET formation in a dose‐dependent manner (Figure 4I), confirming the direct pro‐NET effect of eHSP90α. In vivo, 1G6‐D7 treatment also significantly inhibited NET formation in both CLP and LPS models of sepsis (Figure S7A–H). Together, these findings demonstrate that HSP90α, particularly when released from activated platelets, plays a critical role in promoting NET formation during sepsis.

**FIGURE 5:**
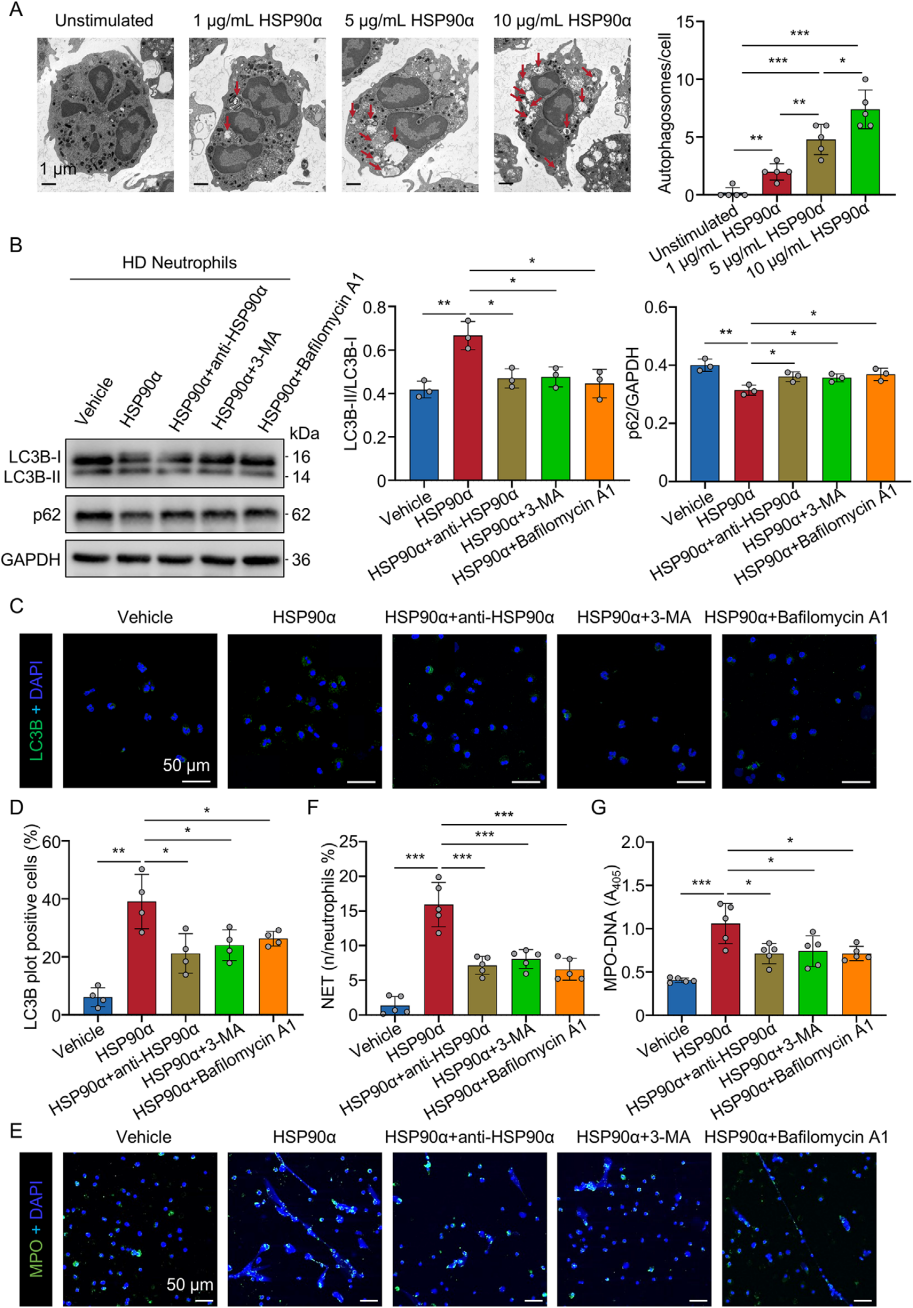
eHSP90α promotes autophagy‐mediated NET formation. (A) Representative images of transmission electron microscope following recombinant HSP90α stimulation (*n* = 5). Scale bar: 1 µm. Red arrowheads denote autolysosomes. Autophagic vesicles (autophagosome/autolysosome) were quantified per cell. (B) Immunoblot and quantification analysis for LC3B and p62 in neutrophils incubated with recombinant HSP90α (10 µg/mL) (*n* = 3) in absence or presence of anti‐HSP90α antibody (10 µg/mL) (*n* = 3), 3‐MA (5 mM) (*n* = 3) and bafilomycin A1 (1 µM) (*n* = 3). (C,D) Representative immunofluorescence microscopy and quantification of LC3B positive cells in neutrophils incubated with recombinant HSP90α (10 µg/mL) (*n* = 4) in absence or presence of anti‐HSP90α antibody (10 µg/mL) (*n* = 4), 3‐MA (5 mM) (*n* = 4) and bafilomycin A1 (1 µM) (*n* = 4). Scale bars: 50 µm. The positive cells were defined as cells with more than five LC3 dots. (E,F), Immunofluorescence and quantification analysis of NETs in neutrophils incubated with recombinant HSP90α (10 µg/mL) (*n* = 5) in absence or presence of anti‐HSP90α antibody (10 µg/mL) (*n* = 5), 3‐MA (5 mM) (*n* = 5) and bafilomycin A1 (1 µM) (*n* = 5). Cells were stained with DAPI for DNA (blue) and anti‐MPO for PMNs or NETs (green). Scale bars: 50 µm. (G) Quantification of MPO‐DNA in the supernatant of neutrophils from each group (*n* = 5). All data are presented as the mean ± SD. Statistical analysis was conducted using one‐way ANOVA and Holm‐Šídák's multiple comparisons test (A–G). ^*^P < 0.05, ^**^P < 0.01, ^***^P < 0.001. Each data point represents one neutrophil (A) or one experiment well (B–G). For A–G, three independent experiments were performed. LC3B, microtubule associated protein 1 light chain 3 beta.

NET formation is known to require autophagy [22]. Analysis of scRNA‐seq also revealed increased expression of autophagy‐related genes and a higher autophagy score in neutrophils from patients with sepsis compared to HD (Figure S8A–C), suggesting that autophagy is actively engaged in neutrophils during sepsis. We also analyzed autophagy in neutrophils and found that LC3B was diffusely distributed in the cytoplasm of HD neutrophils, while in sepsis neutrophils, it appeared as punctate structures, indicating autophagosome formation (Figure S8D). Immunoblot analysis further confirmed a significant increase in the LC3B‐II/LC3B‐I ratio and a concurrent decrease in p62 levels in sepsis neutrophils compared to HD neutrophils, suggesting enhanced autophagic flux (Figure S8E). Additionally, transmission electron microscopy showed that increased accumulation of autophagosomes and autolysosomes in the cytoplasm of neutrophils following recombinant HSP90α treatment compared to unstimulated neutrophils (Figure 5A), indicating elevated autophagic activity in neutrophils driven by recombinant HSP90α stimulation. Incubation of HD neutrophils with recombinant HSP90α resulted in upregulation of LC3B‐II and downregulation of LC3B‐I and p62 compared to unstimulated neutrophils (Figure 5B), consistent with activation of autophagy in neutrophils. This effect was partly reversed by treatment with the inhibitory HSP90α antibody (1G6‐D7), as well as autophagy inhibitors 3‐methyladenine (3‐MA) and bafilomycin A1 (Figure 5B). Confocal microscopy confirmed increased autophagosome formation in HSP90α‐treated neutrophils compared to unstimulated neutrophils, which was effectively blocked by HSP90α antibody, 3‐MA, and bafilomycin A1 (Figure 5C,D). NET formation induced by recombinant HSP90α was significantly suppressed by the HSP90α antibody, 3‐MA, and bafilomycin A1 (Figure 5E–G; Figure S8F). Collectively, these findings indicate that extracellular HSP90α triggers NET formation by inducing autophagy in neutrophils.

### 2.6 eHSP90α Engages TLR4 to Induce Neutrophil Autophagy

**FIGURE 6:**
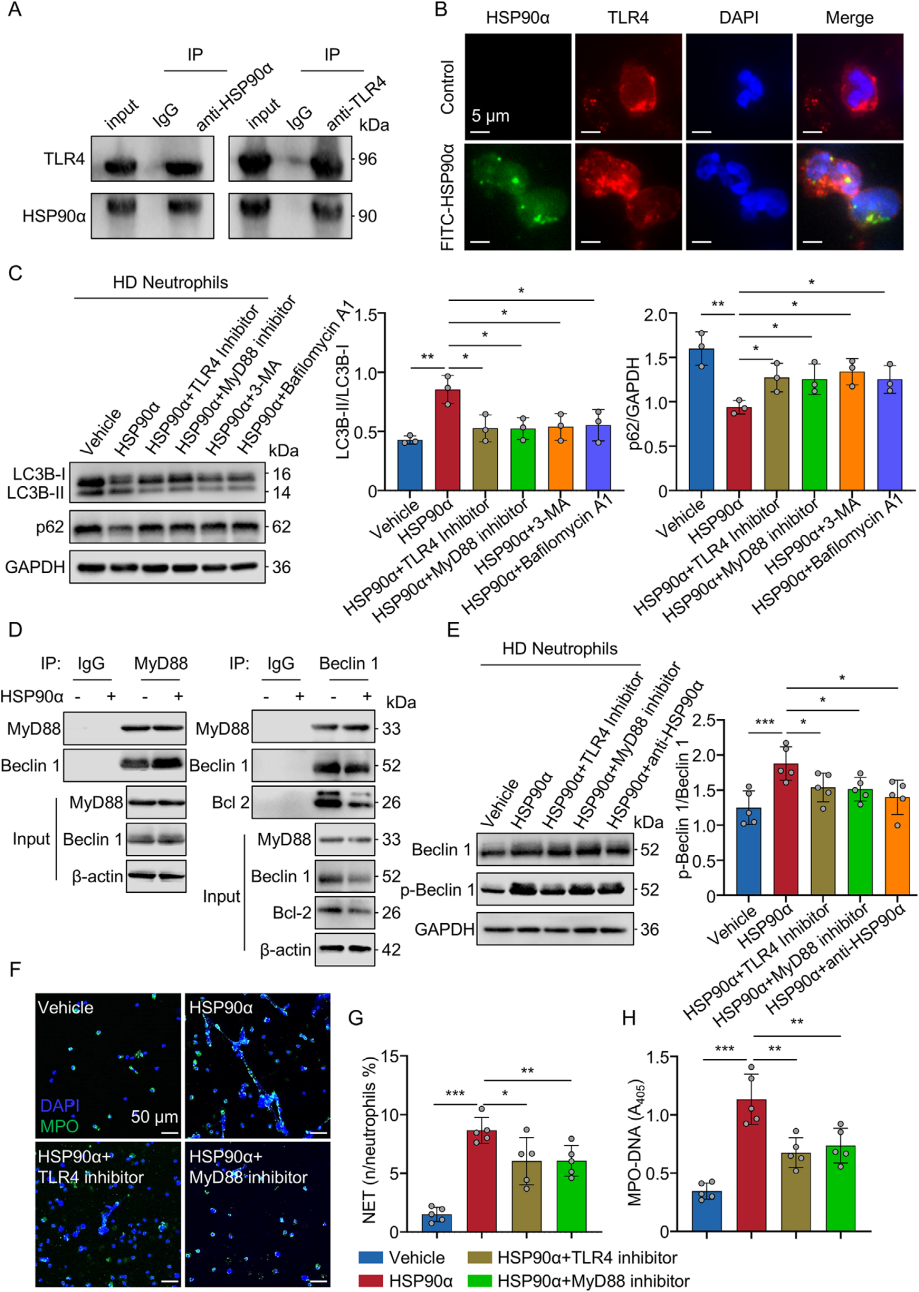
eHSP90α exerts its function through the TLR4‐MyD88 signaling axis. (A) The immunoprecipitation with anti‐HSP90α and anti‐TLR4 antibody was performed in neutrophils. (B) Immunofluorescence analysis of colocalization of FITC‐HSP90α and TLR4 in neutrophils. Control: unlabeled HSP90α. (C) Immunoblot and quantification analysis for LC3B and p62 in neutrophils incubated with recombinant HSP90α (10 µg/mL) (*n* = 3) in absence or presence of TLR4 inhibitor (30 µm) (*n* = 3), MyD88 inhibitor (1 µm) (*n* = 3), 3‐MA (5 mm) (*n* = 3) and bafilomycin A1 (1 µm) (*n* = 3). (D) The immunoprecipitation with anti‐MyD88 and anti‐Beclin 1 antibody was performed in neutrophils incubated with or without recombinant HSP90α (10 µg/mL). (E) Immunoblot and quantification analysis of p‐Beclin 1 and Beclin 1 in neutrophils incubated with recombinant HSP90α (10 µg/mL) (*n* = 5) in absence or presence of anti‐HSP90α antibody (10 µg/mL) (*n* = 5), TLR4 inhibitor (30 µm) (*n* = 5), MyD88 inhibitor (1 µM) (*n* = 5). (F,G) Immunofluorescence and quantification analysis of NETs in neutrophils incubated with recombinant HSP90α (10 µg/mL) (*n* = 5) in absence or presence of TLR4 inhibitor (30 µM) (*n* = 5) and MyD88 inhibitor (1 µM) (*n* = 5). Cells were stained with DAPI for DNA (blue) and anti‐MPO for PMNs or NETs (green). Scale bars: 50 µm. (H) Quantification of MPO‐DNA in the supernatant of neutrophils from each group (*n* = 5). All data are presented as the mean ± SD. Statistical analysis was conducted using one‐way ANOVA and Holm‐Šídák's multiple comparisons test (C, E–H). ^*^ *P* < 0.05, ^**^ *P* < 0.01, ^***^ *P* < 0.001. Each data point represents one experiment well (C, E, G, H). For A–H, three independent experiments were performed. LC3B, microtubule associated protein 1 light chain 3 beta.

To further explore how eHSP90α induces autophagy, we investigated the potential involvement of TLR4, which has been implicated in recognizing other extracellular heat shock proteins [23] but has not been previously linked to this specific pathway in neutrophils. The use of co‐immunoprecipitation assays in neutrophils and 293T cells co‐transfected with Flag‐tagged TLR4 or Flag‐tagged TLR4 extracellular domain and HA‐tagged HSP90α demonstrated a direct interaction between TLR4 extracellular domain and HSP90α (Figure 6A; Figure S9A,B). To further assess the interaction between eHSP90α and TLR4 in HD neutrophils, we also cocultured fluorescently labeled HSP90α (FITC‐HSP90α) with HD neutrophils. Immunofluorescence analysis revealed extensive colocalization between eHSP90α and TLR4 (Figure 6B), indicating a direct interaction at the neutrophil surface.

To assess functional relevance, we treated HD neutrophils with pharmacological inhibitors of TLR4 and its downstream adaptor MyD88. Both inhibitors significantly suppressed HSP90α‐induced autophagic flux, as evidenced by reduced LC3B‐II accumulation and restored p62 expression (Figure 6C). It has been demonstrated that Beclin 1 functions as a client protein of intracellular HSP90, modulating autophagy through its interaction with HSP90 [24]. To determine whether Beclin 1 also contributes to the autophagy of neutrophils led by eHSP90α, we performed protein G/A agarose bead co‐IP assays. In HSP90α‐stimulated neutrophils, both anti‐MyD88 and anti‐Beclin 1 antibodies pulled down higher levels of Beclin 1 and MyD88, respectively, compared to unstimulated cells (Figure 6D; Figure S9C), suggesting enhanced interaction between the two proteins upon stimulation. Furthermore, immunoprecipitation of Beclin 1 from HSP90α‐treated neutrophils revealed a marked reduction in co‐immunoprecipitated Bcl‐2 (Figure 6D; Figure S9C), suggesting disruption of the Beclin 1–Bcl‐2 complex. Importantly, HSP90α stimulation resulted in a significant increase in phosphorylated Beclin 1 (p‐Beclin 1) levels in neutrophils, an effect that was significantly attenuated by the HSP90α‐neutralizing antibody and inhibitors of TLR4 and MyD88 (Figure 6E). Additionally, inhibition of TLR4 or MyD88 significantly reduced NET formation (Figure 6F–H; Figure S9D).

To further validate the role of TLR4 in HSP90α‐induced neutrophil activation, we utilized TLR4^−/−^ mice. Our results showed that HSP90α‐induced autophagy was significantly attenuated in neutrophils isolated from TLR4^−/−^ mice compared to those from WT mice (Figure S10A,B). Recombinant HSP90α protein failed to induce robust NET formation in TLR4^−/−^ neutrophils compared to WT neutrophils (Figure S10C,D). TLR4^−/−^ neutrophils also displayed impaired NET formation in response to sepsis‐derived platelets compared to WT neutrophils, as indicated by a significant reduction in MPO–DNA complexes (Figure S10E,F). Collectively, these findings demonstrate that eHSP90α drives neutrophil autophagy and subsequent NET formation through a TLR4/MyD88/Beclin 1‐dependent signaling pathway.

## 3 Discussion

A significant amount of research has confirmed that patients suffering from sepsis and concomitant DIC exhibit a considerably elevated mortality rate in comparison to those not suffering from DIC [25]. Therefore, treatment is essential to prevent thrombus formation. Platelets and NET are pivotal contributors to immune‐inflammatory thrombosis [26]. However, the interactions between platelets and NET in the context of sepsis require further elucidation. In this study, we demonstrated that HSP90α levels were significantly elevated in septic platelets, a phenomenon attributable to its inheritance from megakaryocytes. Moreover, the release of HSP90α from activated platelets was found to induce autophagy‐driven NET formation through the TLR4/MyD88/Beclin 1 signaling pathway. Consequently, the administration of 1G6‐D7, a neutralizing antibody against HSP90α, effectively inhibited this pathway, resulting in reduced NET formation and attenuation of sepsis‐associated thrombosis and inflammation.

HSP90α, an isoform of HSP90, has been well documented in different cell types of various diseases [27]. However, its function in platelets during sepsis remains poorly understood. Through proteomics analysis, we identified a significant increase in HSP90α expression in platelets from septic conditions. Given that platelets are anucleate cytoplasmic fragments derived from megakaryocytes, and all mRNA species present in platelets are inherited directly from megakaryocytes during thrombopoiesis [28, 29]. Our data suggest that this increase is driven, at least in part, by enhanced trafficking of HSP90α from megakaryocytes into circulating platelets. This is similar to previous findings showing that pro‐inflammatory genes such as *GZMB* and platelet activation‐associated genes like *ITGA2B* are upregulated in megakaryocytes following sepsis, subsequently enriching platelets with these factors [17, 30]. TLR4, a pattern recognition receptor for LPS from Gram‐negative bacteria, is known to play a central role in sepsis [31]. The expression of TLR4 on megakaryocytes was discovered early on [32], but few studies have focused on the function of TLR4 on megakaryocytes. We demonstrate that TLR4 signaling is required for the upregulation of *HSP90α* mRNA expression in megakaryocytes during sepsis, suggesting that megakaryocytes may be involved in a range of responses during sepsis. In addition, it should be recognized that TLR4 expressed on platelets enables them to sense inflammation, and protein synthetic events occur in platelets stimulated with inflammatory agonists [33, 34]. Future studies are warranted to determine whether de novo synthesis of HSP90α also occurs within platelets themselves during sepsis.

HSP90α has been reported to be secreted into the extracellular space by various cell types, particularly tumor cells and keratinocytes [35, 36]. However, whether platelets possess the capacity to secrete HSP90α remains to be elucidated. We demonstrated that upon activation, platelets sequentially mobilize HSP90α to their surface and subsequently release it into the extracellular space. While previous studies on eHSP90α secretion have primarily focused on the exosome‐mediated pathway, recent findings indicating that the exosome‐independent secretory pathway controls 95% of eHSP90α secretion [37]. Consistent with this, we observed the presence of HSP90α released from platelets in both free and exosome‐associated forms. Importantly, we further demonstrated that depletion of platelets significantly—but incompletely—reduced serum HSP90α levels. Endothelial cells have been reported to increase secretion of eHSP90α following activation [38], which may represent an additional source of serum eHSP90α. Anyway, we identified that platelet‐derived HSP90α is a major contributor to the elevated levels of eHSP90α in serum during sepsis.

Intracellular HSP90, a critical molecular chaperone, has been demonstrated to regulate autophagy by interacting with various autophagy‐related proteins. Several proteins involved in autophagy, including Akt [39], Beclin1 [24], Ulk1 [40], and LAMP 2A [41], have been identified as HSP90 client proteins. In addition, a recent study suggests that surface‐expressed HSP90α participates in influenza A virus–induced autophagy by directly binding to viral hemagglutinin [42]. However, the effect of eHSP90α on autophagy has not yet been reported. In our study, we demonstrate that eHSP90α induces autophagy in neutrophils. This process requires MyD88‐mediated recruitment of Beclin 1 from the Beclin 1–Bcl‐2 complex, which may increase the susceptibility of Beclin 1 to phosphorylation by autophagy‐regulating kinases. Consistent with our results, recent studies have demonstrated that MyD88 is a critical mediator of sepsis induced NETs formation [43]. Besides, autophagy plays a crucial role in regulating NET formation [44] and has been shown to prime neutrophils for NET release during sepsis [22]. Here, we demonstrated eHSP90α induced NET formation by triggering TLR4/MyD88/ Beclin 1‐mediated autophagy. These findings provide an insight into how platelet‐derived HSP90α contributes to NET formation during sepsis.

Microvascular thrombosis represents a major challenge in the clinical management of sepsis [45]. Currently, most antithrombotic therapies—such as aspirin and P2Y_12_ inhibitors—function by targeting membrane receptors or intracellular signaling pathways involved in platelet activation [46]. However, the coagulation system in septic patients is characterized by a dynamic imbalance between thrombosis and haemorrhage [47]. As a result, the use of conventional antiplatelet agents can increase the risk of bleeding, particularly in patients with sepsis‐associated thrombocytopenia [46, 48]. Therefore, alternative strategies that target pathogenic platelet‐derived factors rather than platelet activation per se may offer a safer and more effective therapeutic approach. In this study, we identified that 1G6‐D7 effectively suppresses NET formation induced by septic platelets and mitigates thrombosis, suggesting a promising therapeutic strategy in thrombosis‐related diseases. However, during clinical translation, consideration must be given to the potential off‐target effects of 1G6‐D7, as HSP90α is a widely expressed protein. Consequently, the precise administration of 1G6‐D7 is imperative to circumvent any potential inhibition of HSP90α function in healthy cells.

However, the present study does have several limitations. As sepsis progresses from the early to the middle and late phases, platelets undergo dynamic changes—from hyperactivation to subsequent dysfunction following extensive granule release [49]. Given this temporal heterogeneity, the relatively small cohort size may not fully capture the spectrum of platelet proteomic and cytokine alterations across different phases of sepsis. Future studies with larger, stage‐stratified cohorts will be necessary to validate and extend these findings. Moreover, the clinical samples utilized in this study were exclusively from children. Research indicates that, although platelet function in children may resemble that of adults after the age of one, differences persist, such as reduced granule release, diminished expression of integrin αIIbβ3, and impaired GPIα internalization [50]. Consequently, it is imperative to exercise caution when directly extrapolating our findings to adult sepsis. In addition, the therapeutic application of HSP90α neutralization must be approached with caution. Although our data demonstrate that anti‐HSP90α antibody treatment effectively mitigates thrombosis and inflammation in vivo, systemic inhibition of HSP90 could potentially interfere with the intracellular chaperone functions of HSP90, which are essential for protein folding, stress responses, and cellular homeostasis. Furthermore, since HSP90α shares a high degree of sequence similarity with HSP90β, off‐target effects on other isoforms or unintended immune reactions may occur. The pharmacokinetics, tissue distribution, and long‐term safety of anti‐HSP90α antibodies therefore require rigorous preclinical evaluation before translation to clinical settings.

Species‐ and age‐related differences between pediatric human samples and adult mouse models are important considerations when interpreting our findings. Mice and humans differ markedly in platelet count, receptor density, and co‐receptor expression, which may affect platelet–neutrophil communication and immune activation. Furthermore, the immune phenotype of specific pathogen‐free (SPF) laboratory mice has been reported to resemble that of newborn rather than adult humans [51], suggesting that murine models more closely approximate certain aspects of pediatric immunity. Despite these differences, both systems demonstrate that platelet‐derived HSP90α promotes NET formation, supporting a conserved mechanism of platelet–neutrophil interaction across species.

In conclusion, our findings highlight the critical role of platelet‐derived eHSP90α in driving autophagy‐dependent NET formation and propose that targeting eHSP90α with a specific antibody may represent a potentially promising treatment for septic thrombosis.

## 4 Materials and Methods

### 4.1 Patients Study

We recruited 20 patients (17 with sepsis without shock and 3 with septic shock) and 20 age‐matched healthy donors (HD), from Children's Hospital Affiliated to Zhengzhou University, China, between June 2023 and December 2023. Sepsis was diagnosed based on 2005 international pediatric sepsis consensus conference: definitions for sepsis and organ dysfunction in pediatrics. The diagnostic criteria for sepsis are: systemic inflammatory response syndrome (SIRS) in the presence of or as a result of suspected or proven infection. The diagnostic criteria for septic shock are: sepsis and cardiovascular organ dysfunction. This cohort consists entirely of children (mean age ∼4 years), and the study population is shown in Table S1. Peripheral blood samples were obtained on day 1 when these patients were newly diagnosed with sepsis upon ICU admission. Peripheral blood samples of HD were obtained from the individuals undergoing routine medical examinations. Informed consent was obtained from the patients or their families, and the study was approved by the Medical Ethics Committee of the Children's Hospital Affiliated to Zhengzhou University (Approval No: 2024‐KY‐0075‐002).

### 4.2 Animal Treatment

C57BL/6 mice were obtained from the Laboratory Animal Center of Zhengzhou University. TLR4 knockout (TLR4^−/−^) mice were obtained from Cyagen Biosciences. All mice were housed in the Laboratory Animal Center of Zhengzhou University at a constant temperature of 18–22°C and a humidity of 55%–60%, with a 12:12 h light/dark cycle. Procedures were approved by the Institutional Animal Care and Use Committee of Zhengzhou University (ZZU‐LAC20250620[23]). All mouse experiments complied with the principles of animal protection, welfare, ethics, and 3R (replacement, reduction, and refinement).

To establish a murine sepsis model, the cecum was ligated at the indicated site to achieve the desired severity, and a single penetrating puncture of the ileocecal valve was performed with a 19‐gauge needle. Mice that underwent the sham procedure were subjected to the same procedure except for cecal ligation and perforation. For platelet depletion, 4 µg/kg platelet‐depleting antibody (Emfret Analytics, R300) was administered via the tail vein daily following CLP. For the anti‐HSP90α antibody group, 5 mg/kg of anti‐HSP90α antibody (1G6‐D7, Merck, MABC1106) was administered via the tail vein daily following CLP. For the toxicity detection of anti‐HSP90α antibody, 5 mg/kg of anti‐HSP90α antibody (1G6‐D7, Merck, MABC1106) or control was administered via the tail vein daily. For the HSP90α group, 500 ng/mouse of recombinant HSP90α (Cayman, 22734) was administered via the tail vein once every two days following CLP. For neutrophil depletion, 200 µg/mouse of anti‐Ly6G antibody (Bioxcell, BE0075‐1) was intraperitoneally injected once every two days following CLP. After a period of five days, all mice were euthanized, and the serum, lung, and liver tissues were collected for further analysis.

To build the LPS‐induced sepsis model, 5‐week‐old mice were intraperitoneally injected with LPS (10 mg/kg; Sigma, L4391) for 24 h. For platelet depletion, mice were intravenously injected with platelet‐depleting antibody (Emfret Analytics, R300) one day before LPS administration. For anti‐HSP90α antibody intervention, mice were intravenously injected with 1G6‐D7 (Merck, MABC1106) immediately after LPS administration. For the HSP90α group, 500 ng/mouse of recombinant HSP90α (Cayman, 22734) was administered via the tail vein following LPS administration. For neutrophil depletion, 200 µg/mouse of anti‐Ly6G antibody (Bioxcell, BE0075‐1) was intraperitoneally injected one day before LPS administration.

### 4.3 Isolation of Human Platelets and Platelet‐derived Exosomes

Venous blood samples were collected from HD and septic patients using blood collection tubes containing 3.8% trisodium citrate. The blood sample was centrifuged at 250 g for 15 min at 25°C to collect platelet‐rich plasma (PRP). PRP was then treated with 100 nm prostaglandin E1 (MCE, HY‐B0131) and centrifuged at 1000 g for 5 min. The collected supernatant is platelet‐poor plasma (PPP), and the platelet precipitate was washed and resuspended with Advanced Tyrode's Solution containing HEPES (Pricella, PB180340). For isolation of exosome, the platelet‐enriched pellet was resuspended in Advanced Tyrode's Solution and subsequently centrifuged at 4000 g for 10 min at 4°C to precipitate platelets. The supernatant was incubated with Total Exosome Isolation (Invitrogen, 4 478 359) for 60 min at 4°C and then centrifuged at 10 000 for 30 min at 4°C to collect the precipitated exosome.

### 4.4 Isolation of Human and Mouse Neutrophil

Peripheral blood samples were collected from human and mouse subjects using blood collection tubes or solutions containing 3.8% trisodium citrate. Human and mouse neutrophils were obtained with the Human Peripheral Blood Neutrophil Isolation Kit (Solarbio, P9040) and Mouse Peripheral Blood Neutrophil Isolation Kit (Solarbio, P9201), according to the manufacturer's instructions.

### 4.5 Isolation of Murine Platelets and Bone Marrow Megakaryocytes

Blood was collected by cardiac puncture in anaesthetized mice into tubes containing 3.8% trisodium citrate, and mouse platelets were isolated using the same method as for human platelets. For isolation of murine bone marrow megakaryocytes, femurs from control and 3‐day post‐CLP mice were rinsed with sterile PBS and centrifuged at 1200 rpm for 5 min to collect the precipitate, and erythrocytes were removed with erythrocyte lysate (Solarbio, R1010). Bone marrow cells were incubated with anti‐CD41 antibody for 20 min at 4°C, protected from light, and then sorted by flow cytometry on a FACS Aria cell sorter.

### 4.6 Proteomic Sequencing of Platelets

The isolation of human platelets was subjected to high‐throughput proteomics sequencing by PTM BIO (Hangzhou, China). Each group of samples was added to four times the volume of lysis buffer (1% SDC, 1% protease inhibitor), heated at 95°C for 20 min, and sonicated. The samples were subjected to a centrifugal process at a temperature of 4°C and an angular velocity of 12 000 g for a duration of 10 min. Thereafter, the resultant upper layer was transferred to a fresh centrifuge tube. Protein concentration was determined by means of a BCA kit. For digestion, the protein solution was reduced to 5 mm dithiothreitol for 30 min at 56°C and alkylated with 11 mm iodoacetamide for 15 min at room temperature in the dark. The protein sample was then diluted by adding 200 mm TEAB to the urea concentration less than 2 m. Finally, trypsin was added at 1: 50 trypsin‐to‐protein mass ratio for the first digestion overnight and 1:100 trypsin‐to‐protein mass ratio for a second 4 h digestion. Finally, the peptides were desalted by a Strata X SPE column. The tryptic peptides were dissolved in solvent A, directly loaded onto a home‐made reversed‐phase analytical column (25‐cm length, 100 µm i.d.). The mobile phase consisted of solvent A (0.1% formic acid, 2% acetonitrile/in water) and solvent B (0.1% formic acid in acetonitrile). Peptides were separated with following gradient: 0–14 min, 6%–24% B; 14–16 min, 24%–35% B; 16–18 min, 35%–80% B; 18–20 min, 80% B, and all at a constant flow rate of 500 nL/minon a NanoElute UHPLC system (Bruker Daltonics). The peptides were subjected to a capillary source followed by the timsTOF Pro 2 mass spectrometry. The electrospray voltage applied was 1.75 kV. Precursors and fragments were analyzed at the TOF detector. The timsTOF Pro was operated in data‐independent parallel accumulation serial fragmentation (dia‐PASEF) mode. The full MS scan was set as 300–1500 (MS/MS scan range) and 20 PASEF (MS/MS mode) ‐MS/MS scans were acquired per cycle. The MS/MS scan range was set as 400–850, and the isolation window was set as 7 m/z.

### 4.7 Data‐independent Acquisition (DIA) Data Analysis

The DIA data was processed using DIA‐NN (v1.8.1). Tandem mass spectra were searched against Homo_sapiens_9606_SP_20231220.fasta (20429 entries), concatenated with a reverse decoy database. Trypsin/P was specified as a cleavage enzyme allowing up to 1 missing cleavage. Excision on N‐terminal Met and carbamidomethyl on Cys were specified as fixed modifications. FDR was adjusted to < 1%. DIA‐NN normally reported precursor levels were exported, and downstream data were log‐transformed. Take the log2 of the relative intensity value to make it fit a normal distribution, and then use an unpaired two‐tailed t‐test to calculate the differentially expressed proteins. Criteria of fold change > 1.5 and P < 0.05 were used to determine statistically significant differences. The identified proteins from platelets were annotated and classified into pathways using the Reactome database (2016 version).

Raw data from the dataset OMIX001255 were downloaded and processed using DIA‐NN (v1.8.1) as in the previous study. Briefly, the human protein sequence database downloaded from Uniprot was loaded into DIA‐NN, and a specific spectral library was generated. The protein of OMIX001255 was analyzed qualitatively and quantitatively by comparison with the spectral library. DIA‐NN normally reported precursor levels were exported, and differentially expressed proteins (DEPs) were identified using the Bayesian analysis in the Limma package. Log2 fold change > 1, and P < 0.05 were used to determine statistically significant differences.

### 4.8 Public Single‐cell RNA Sequencing Data Analysis

Single‐cell RNA‐seq data from GSE167363 were downloaded and processed in R using Seurat (v4.3.0). Cells with > 10% mitochondrial gene expression or < 200 detected genes were excluded from downstream analysis. Doublets were identified and removed using Doublet Finder (v2.0.4). Gene expression matrices were log‐normalized, and 2000 highly variable genes were selected using Find Variable Features. Prior to dimensionality reduction, expression values were scaled and centered using Seurat's Scale Data function. Sample integration and batch correction were performed using Harmony (v1.2.0). Principal component analysis (PCA) was then run on the scaled, integrated assay, and the top 30 principal components (PCs) were used for downstream analyses, including dimensionality reduction, clustering, and visualization.

Differential abundance (DA) was assessed using MiloR (v2.5.1), with kNN graph construction (k = 30, 30 Harmony PCs), refined neighborhood detection, sample‐wise neighborhood counting, and DA testing using a linear model. Neighborhoods were annotated to cell types, and results were visualized with bee swarm plots.

Autophagy activity was quantified using Seurat's Add Module Score function based on a predefined autophagy gene module (ATG3, ATG4A, ATG4B, ATG4C, ATG4D, ATG5, ATG7, ATG9A, ATG10, ATG12, ATG13, ATG14, BECN1, MAP1LC3A, MAP1LC3B). Differences in autophagy scores between different groups were assessed using the Wilcoxon rank‐sum test.

### 4.9 Detection of Platelet HSP90α by Immunofluorescence

For HD or sepsis, isolated platelet‐rich plasma (PRP) was fixed in 4% paraformaldehyde (Servicebio, G1101) for 15 min and centrifuged at 950 g for 11 min. The platelet precipitate was washed twice with Advanced Tyrode's Solution and seeded onto poly‐L‐lysine‐coated coverslips (Sigma, 602895). The platelets were left at 37°C for 90 min to allow adhesion. Platelets were blocked with Protein Free Rapid Blocking Solution (Servicebio, G2052) for 10 minutes at room temperature and permeabilized with 0.2% Triton X‐100 (Servicebio, G3068). Platelets were stained with anti‐HSP90 alpha antibody (Abcam, ab79849) and anti‐CD41 antibody (Abcam, ab134131) overnight at 4°C, followed by Alexa Fluor 488‐labeled goat anti‐rabbit IgG (Servicebio, GB25303) and Cy3‐labelled donkey anti‐mouse IgG (Servicebio, GB21401). For the platelets stimulated with thrombin, isolated PRP was treated with thrombin (0.05 U/mL) or a combination of latrunculin A (200 µm) and thrombin (0.05 U/mL) for 10 min prior to fixation and centrifugation. For the platelets stimulated with HD PPP or sepsis PPP, washed platelets were suspended in Advanced Tyrode's Solution containing 10% HD PPP or sepsis PPP for 10 min prior to fixation and centrifugation. Immunofluorescence confocal images were obtained using Leica THUNDER Imaging Systems and Laser Scanning Confocal Microscope LSM980.

### 4.10 Detection of Platelet HSP90α by Flow Cytometry

To detect HS90α on the membrane surface, isolated platelets were stimulated with thrombin (0.05 U/mL) and incubated with mouse anti‐HS90α primary antibody (Abcam, ab79849) for 20 min in the dark, washed, and then incubated with an appropriate Cy3‐conjugated secondary antibody (Servicebio, GB21401) for one hour. For the intracellular HS90α detection, resting and stimulated‐platelets were fixed and permeabilized by BD Cytofix/CytopermTM Fixation/Permeabilization Kit (BD Bioscience, 554714). Then, platelets were incubated with anti‐HS90α primary antibody (Abcam, ab79849) and a subsequent secondary antibody. For HD and patients with sepsis, HSP90α expression was analyzed in washed platelets following the intracellular labeling with HSP90α. For the platelets stimulated with HD PPP or sepsis PPP, washed platelets were suspended in Advanced Tyrode's Solution containing 10% HD PPP or sepsis PPP for 10 min, followed by detection of HS90α expression on the platelet surface and intracellular. For the platelets stimulated with microvesicles/exosomes‐depleted PPP, microvesicles and exosomes in PPP were removed by ultracentrifugation (100000 g for 60 min at 4°C), and washed platelets were suspended in Advanced Tyrode's Solution containing 10% microvesicles/exosomes‐depleted PPP from HD or sepsis for 10 min, followed by detection of HS90α expression on the platelets' surface and intracellular. All samples were analyzed using BD FACSAria II and data processed using FlowJo (v. 10.6.1).

### 4.11 Detection of Platelet‐Released HPS90α

For the detection of platelet‐released HPS90α, isolated platelets were adjusted to a density of 1 × 10^9^ cells/mL and were stimulated with thrombin (0.05 U/mL) at 37°C for the indicated time. The pellet and releasate were separated by centrifugation at 5000 g for five min. The protein in the pellet was extracted by RIPA Lysis Solution (Epizyme, PC101), and the protein in the releasate was condensed by methanol‐chloroform. The protein in pellet and releasate was subsequently analyzed by immunoblotting. Furthermore, the concentration of HSP90α in the releasate was measured by HSP90α ELISA Kit (Enzo, ADI‐EKS‐895). For detection of the exosome‐associated HSP90α release, isolated platelets were stimulated with thrombin (0.05 U/mL) or thrombin (0.05 U/mL) combined with GE4869 (20 µm) (Merck, D1692) for 6 h. Then, the concentration of HSP90α in the supernatant was measured by HSP90α ELISA Kit (Enzo, ADI‐EKS‐895).

### 4.12 NET Formation Experiment

For NET formation in peripheral neutrophils, isolated neutrophils were cultured in 1640 medium (Procell, PM150110A) supplemented with 10% fetal bovine serum (Procell, 164220) for 4 h in a CO_2_ incubator. For PPP‐induced NET formation, isolated HD neutrophils were cultured for 4 h in 1640 medium supplemented with 10% HD PPP, sepsis PPP, or a mixture of sepsis PPP and anti‐HSP90α antibody (Merck, MABC1106). For NET formation in human platelet‐neutrophil co‐culture, thrombin (Absin, abs47047374) pre‐activated HD and sepsis platelets (0.04 × 105/ml) were seeded on poly‐L‐lysine‐coated coverslips. After 15 min, HD neutrophils (2 × 105/ml) were added and incubated for 4 h with or without HSP90α antibody. For HSP90α‐induced NET formation, isolated HD neutrophils were cultured with 1, 5, or 10 µg/mL recombinant HSP90α (Cayman, 22734). For autophagy‐mediated NET formation, isolated HD neutrophils were cultured with 10 µg/mL recombinant HSP90α, or supplemented with 10 µg/mL anti‐HSP90α antibody, 5 mm 3‐MA (MCE, HY‐19312), or 1 µm Bafilomycin A1 (MCE, HY‐100558). For TLR4‐mediated NET formation, isolated HD neutrophils were cultured with 10 µg/mL recombinant HSP90α, or supplemented with 30 µm TLR4 inhibitor VIPER (Novus Biologicals, NBP2‐26244) or 1 µm MYD88 inhibitor MyD88‐IN‐1 (MCE, HY‐149992). 3‐MA and TLR4 inhibitor VIPER were dissolved with PBS. Bafilomycin A1 and MYD88 inhibitor MyD88‐IN‐1 were dissolved with dimethyl sulfoxide (DMSO) (MCE, HY‐Y0320C). To remove the influence of DMSO, an equal volume of DMSO was added to the vehicle group and other groups not containing DMSO.

The NET formation was quantified by confocal microscopy and the relative content of MPO‐DNA complexes in supernatants. Briefly, the neutrophils were fixed and stained with anti‐MPO antibody (Abcam, ab208670), Alexa Fluor 488‐labeled goat anti‐rabbit IgG (Servicebio, GB25303) and DAPI (Servicebio, G1012). MPO‐DNA complexes in media supernatants were captured with a polyclonal anti‐MPO primary antibody (Usbiological, 525712) and detected with a peroxidase‐labelled anti‐DNA primary antibody (Roche, 11774425001) according to the manufacturer's protocol.

### 4.13 Detection of Autophagy in Neutrophils

The autophagy in neutrophils was detected by confocal microscopy and transmission electron microscopy (TEM). Briefly, isolated neutrophils or HSP90α‐stimulated neutrophils were fixed and stained with anti‐LC3B (Abcam, ab192890), Alexa Fluor 488‐labelled goat anti‐rabbit IgG (Servicebio, GB25303), and DAPI (Servicebio, G1012). For TEM, the neutrophils were fixed in an electron microscope fixative (Servicebio, G1102), dehydrated using alcohol, embedded in epoxy resin, and sectioned at a thickness of 1.5 µm. Sections were subjected to staining with uranyl acetate and lead citrate, and were then examined using a Hitachi HT7700 transmission electron microscope (Tokyo, Japan).

### 4.14 Detection of the Interaction Between HSP90α and TLR4

For exogenous immunoprecipitation (IP), the plasmid pCDNA3.1‐HA‐HSP90α pCDNA3.1‐Flag‐TLR4 and pCDNA3.1‐Flag‐TLR4 extracellular domain (24‐631 aa) were built and transfected into 293T cells from American Type Culture Collection (ATCC) as described in the figures. Cells were washed and scraped into ice‐cold PBS, then lysed with Lysis Buffer for IP (Epizyme, PC105) for 30 min on ice. The cells were centrifuged at 4°C, 12000 rpm for 10 min, and the supernatant was collected. As an input control, approximately one‐fifth of the protein supernatant was retained. The lysates were incubated with anti‐FLAG M2 affinity beads (Merck, M8823) or anti‐HA Magnetic Beads (Beyotime, P2121) overnight at 4°C. The beads were collected by a magnetic rack. The prey proteins were eluted from magnetic beads by boiling in SDS buffer, and were subsequently analyzed by WB.

For endogenous IP, isolated neutrophils were treated with recombinant HSP90α (10 µg/mL) for 2 h, followed by lysis with Lysis Buffer for IP (Epizyme, PC105). The cell lysates were incubated with corresponding antibody against the target protein at 4°C overnight. The following day, lysates were incubated with 25 µL of protein A/G agarose beads (Epizyme, YJ201) for 6 h at 4°C. The antigen‐antibody‐bead complexes were collected by magnetic rack, after which the prey proteins were eluted by boiling in SDS buffer and analyzed by WB. The expression levels of the prey proteins were then normalized with the β‐actin. Antibodies used in IP included anti‐TLR4 antibody (Abcam, ab8376 and Proteintech, 19811‐1‐AP), anti‐HSP90α antibody (Abcam, ab2928 and Abcam, ab79849), anti‐MyD88 antibody (Abcam, ab219413 and CST, 50010), anti‐Beclin 1 (Abcam, ab207612 and CST, 4122) anti‐Bcl 2 antibody (HUABIO, ET1702‐53). To avoid interference from IgG heavy and light chains, primary antibodies of different species origins were employed for IP and WB analyses. Furthermore, during WB detection, Anti‐mouse IgG for IP (Vazyme, RA1009‐01) or Anti‐rabbit IgG for IP (Vazyme, RA1008‐01) were utilized.

For the immunofluorescence of HSP90α‐stimulated neutrophils, FITC‐modified recombinant HSP90α was synthesized by Sangon Biotech and treated with the neutrophils for a period of 30 minutes. The neutrophils were subjected to fixation and staining with an anti‐TLR4 antibody (Abcam, ab22048), followed by Cy3‐labeled donkey anti‐mouse IgG (Servicebio, GB21401) and DAPI.

### 4.15 Detection of HSP90α mRNA Expression in Platelet and Megakaryocyte

The extraction of platelet and megakaryocyte mRNA was conducted using TRIzol, followed by reverse transcription into cDNA templates with the PrimeScript RT Reagent Kit with gDNA Eraser (Perfect Real Time) (TaKaRa, RR047Q), in accordance with the manufacturer's protocol. All quantitative real‐time polymerase chain reaction (q‐PCR) experiments were performed utilising FastSYBR Mixture (CWBIO, CW0955M) and detected using the Rotor Gene 3000. For each gene tested, GAPDH expression was used as an internal control, and the 2(‐ΔΔCt) method was used to quantify the relative mean fold change and propagate the standard error. The forward and reverse primers for HSP90AA1 (human) were as follows: 5'‐AGGAGGTTGAGACGTTCGC‐3' and 5'‐AGAGTTCGATCTTGTTTGTTCGG‐3'. The primers for Hsp90aa1 (mouse) were forward 5'‐AATTGCCCAGTTAATGTCCTTGA‐3' and reverse 5'‐CGTCCGATGAATTGGAGATGAG‐3'.

For the isolated megakaryocytes experiment, the samples were obtained from wild‐type (WT) and TLR4^−/−^ mice. These megakaryocytes were then cultured in 1640 medium supplemented with PPP from WT sham or WT cecum ligation and puncture (CLP) mice. This culture was performed for a period of 2 hours. Subsequently, the expression of HSP90a mRNA was detected.

For the dataset PRJNA521077, the raw data were downloaded, and the quality was checked. The fragments per kilobase of exon per million reads (FPKM) were then quantified. Differential expression and significance of HSP90AA1 were determined using the DESeq2 algorithm.

### 4.16 Western Blotting

Western blotting techniques were used to detect proteins extracted from human platelets and neutrophils. Protein from platelets and neutrophils was extracted with RIPA Lysis Solution (Epizyme, PC101), separated by SDS‐PAGE, and transferred to PVDF membranes by wet transfer. The membranes were incubated with primary and secondary antibodies and exposed to ECL chemiluminescence solution (Thermo, 34579). The resulting images were processed using Image Lab (v.6.1.0). Relative protein expression was quantified using Imaga J (v.1.8.0). Antibodies used in this study include HSP90α (Abcam, ab79849), GAPDH (Abcam, ab8245), LC3B (Abcam, ab192890), p62 (Abcam, ab207305), anti‐MyD88 (Abcam, ab219413), Beclin 1 (Abcam, ab207612), Bcl‐2 (Abcam, ab182858), Phospho‐Beclin‐1 (Ser93) (CST, 14717) and goat anti‐rabbit IgG H&L (HRP) (Abcam, ab205718).

### 4.17 Enzyme‐linked Immunosorbent Assay (ELISA)

The levels of HSP90α (Biorbyt, orb1173524), thrombin‐antithrombin (TAT) complex (Siemens, OWMG15), TNF‐α (BD Pharmingen, 555268), IL‐6 (BD Pharmingen, 555240), and IL‐1β (BOSTER, EK0392) in mouse plasma or HSP90α (Enzo, ADI‐EKS‐895) in human plasma were detected using ELISA kits according to the manufacturer's instructions. MPO‐DNA complexes in human/mouse plasma and media supernatants were captured with a polyclonal anti‐MPO primary antibody (Usbiological, 525712) and detected with a peroxidase‐labelled anti‐DNA primary antibody (Roche, 11774425001) according to the manufacturer's protocol.

### 4.18 Transmission Electron Microscopy (TEM)

The TEM analysis was conducted by Servicebio (Wuhan, China). Briefly, the cells were fixed in an electron microscope fixative (Servicebio, G1102), dehydrated using alcohol, embedded in epoxy resin, and sectioned at a thickness of 1.5 µm. Sections were subjected to staining with uranyl acetate and lead citrate, and were then examined using a Hitachi HT7700 transmission electron microscope (Tokyo, Japan).

### 4.19 Histology

Fresh liver and lung tissue were fixed in 4% paraformaldehyde (Servicebio, G1101), dehydrated with alcohol, embedded in paraffin, and cut into 4 µm‐thick pathological slices. For H and E staining, the slice was stained with hematoxylin and eosin (Servicebio, G1005). For IHC staining, the slices were subjected to antigen retrieval and incubated with anti‐CD41 antibody (Abcam, ab134131), HRP‐labelled secondary antibodies (Abcam, ab150077), and DAB substrate (Servicebio, G1212). Dual fluorescent staining was employed to detect the NET formation in the liver and the lung. Briefly, the slice was treated with an anti‐MPO antibody (Abcam, ab208670), followed by sequential incubation with secondary antibodies and iF488‐Tyramide dye (Servicebio, G1231). After antigen repair again, the anti‐Histone H3 (citrulline R2 + R8 + R17) antibody (Abcam, ab281584) was incubated, followed by sequential incubation with secondary antibodies and iF647‐Tyramide dye (Servicebio, G1232). Nuclei were stained with DAPI (Servicebio, G1012). Immunofluorescence images were acquired with Leica THUNDER Imaging Systems (Wetzlar, Germany).

### 4.20 Statistical Analysis

The experimental data were subjected to statistical analysis and graphical representation using GraphPad Prism (v.9.5.1), with continuous variables expressed as mean ± standard deviation (SD). Comparisons between two groups were analyzed using an unpaired non‐parametric Student's t‐test, while comparisons between more than two groups were analyzed using a one‐way analysis of variance (ANOVA) followed by Holm–Šídák's test. Pearson's correlation test was used to analyze the linear relationship between two variables. Statistically speaking, a *P* value of 0.05 or less was deemed to be significant.

## Author Contributions

W.‐C.B., L.‐M.D., and S.‐C.C. contributed equally to this work and also co‐first authors. Study conceptualization: W.‐C.B., Z.‐X.Z. and L.‐Z.L.; Methodology: Z.‐X.Z., S.‐C.C., and L.Y.; Formal analysis and investigation: L.‐Z.L., C.‐J.Y., L.‐L.F., J.‐Y.Y., and H.‐Y.S.; Writing – original draft preparation: W.‐C.B. and C.‐L.D.; Writing – review and editing: L.‐Z.L., and Z.‐X.Z.; Writing‐spell check: Z.‐Y.D., L.‐Y.C., Z.‐C.L., M.‐Y.L., Z‐Y.X., and L.‐N.Y.; Funding acquisition: C.‐Y.B., C.‐L.D., and Z.‐X.Z.; Supervision: L.‐Z.L., Z.‐X.Z., and W.‐C.B. All authors read and approved the final paper.

## Conflicts of Interest

The authors declare no conflict of interest.

## Supporting information




**Supporting File 1**: advs74491‐sup‐0001‐SuppMat.docx.

**Supporting File 2**: advs74491‐sup‐0002‐DataFile.xlsx.

## Data Availability

The data that support the findings of this study are available on request from the corresponding author. The data are not publicly available due to privacy or ethical restrictions.
