# Supplementary material for: Activated Platelet–Released Heat Shock Protein 90α Triggers Autophagy‐Dependent Neutrophil Extracellular Trap Formation and Amplifies Sepsis
Source: Adv Sci (Weinh). 2026 Feb 24;13(25):e15933. doi: 10.1002/advs.202515933 (PMC13137805; doi:10.1002/advs.202515933)
Supplement: Supplementary file 1 — Supporting File 1: advs74491‐sup‐0001‐SuppMat.docx. [file ADVS-13-e15933-s002.docx]

**
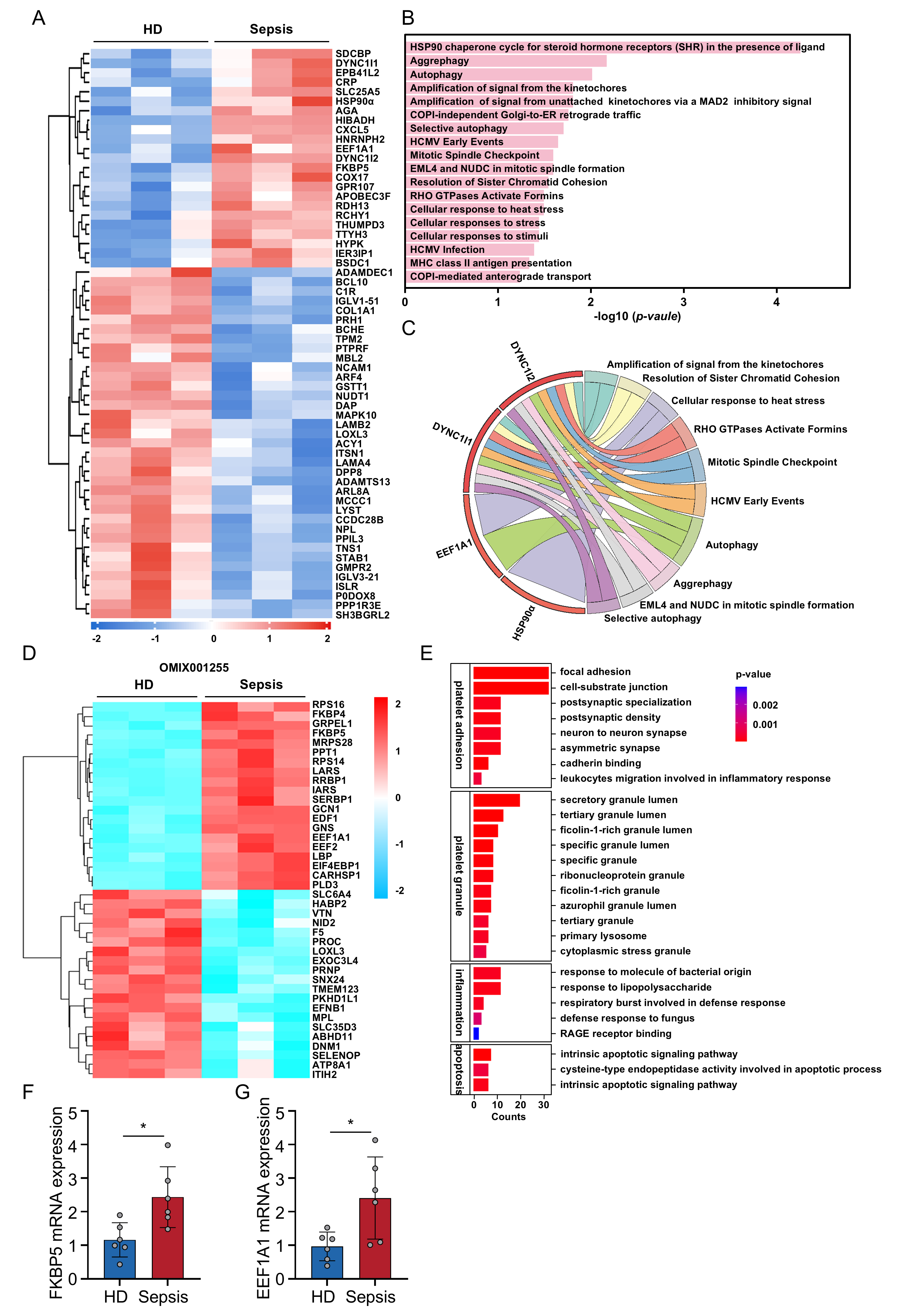
Figure S1.** **Proteomic analysis reveals that platelets are activated in patients with sepsis.** **(A)** Heatmap with significantly differential expression of proteins from the HD and sepsis groups. Cutoff: fold change > 1.5 and *P* value < 0.05. **(B)** Bar plots of the enriched Reactome terms of highly expressed genes from sepsis groups. **(C)** Chordal graph of the enriched Reactome terms of highly expressed genes from sepsis groups. **(D)** Heatmap with top 20 significantly differentially expressed proteins in HD and sepsis groups from OMIX001255. Cutoff: fold change > 2 and *P* value < 0.05. **(E)** Bar plots of the enriched GO biological processes of highly expressed proteins in sepsis groups from OMIX001255. **(F** and **G)** FKBP5 and EEF1A1 mRNA was quantified in platelets from HD (*n* = 6) and sepsis patients (*n* = 6). All data are presented as the mean ± SD. Statistical analysis was conducted using an unpaired two-tailed *t*-test (F, G). **P* < 0.05.

**
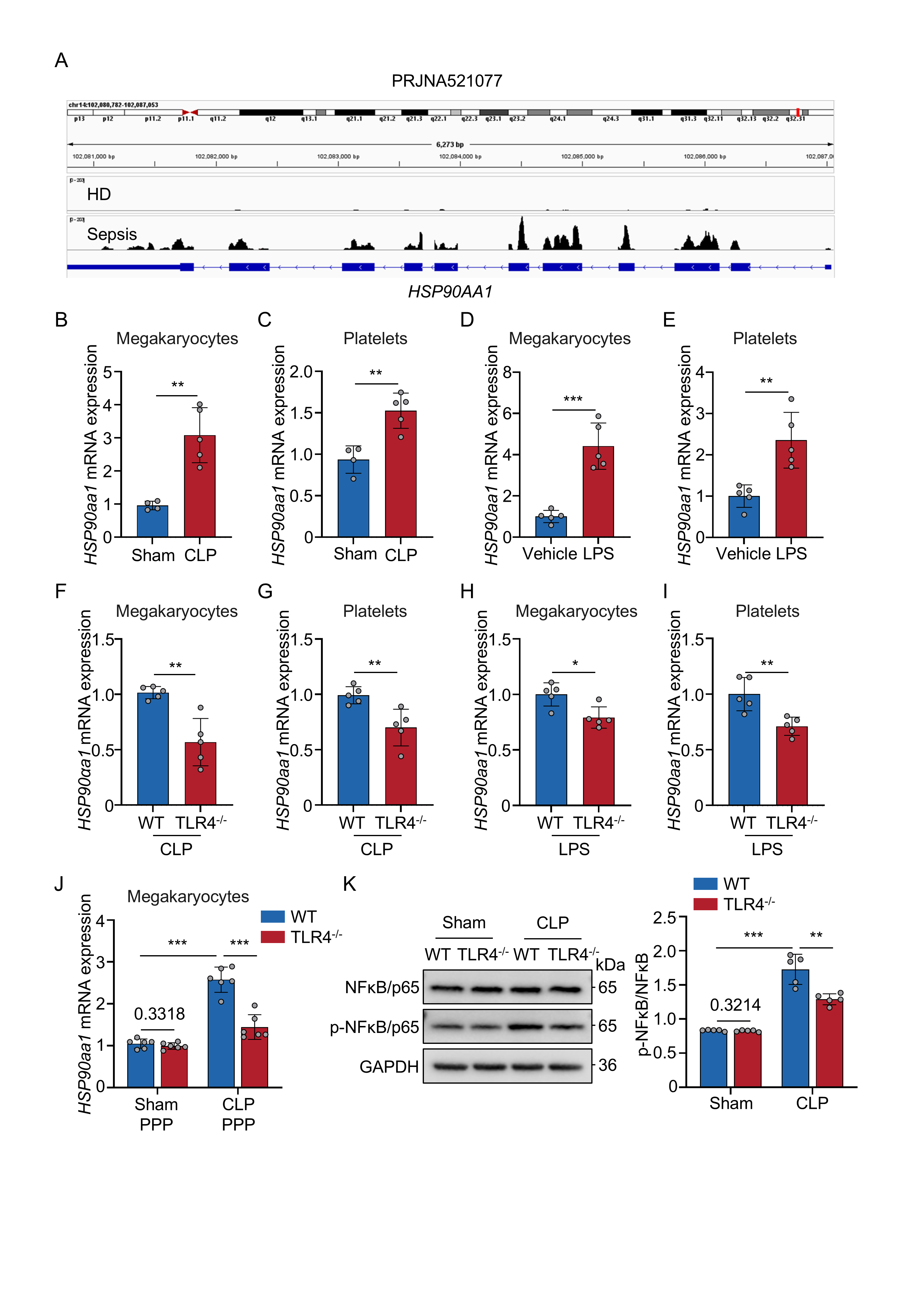
Figure S2.** **Elevated expression of HSP90α mRNA ensues in platelets from the mouse model of sepsis.** **(A)** Integrated Genomics Viewer (IGV) image of *HSP90AA1* exonal reads in platelets of a healthy subject (top) and a sepsis patient (bottom) from publicly available RNA-seq data (PRJNA521077). **(B** and **C)** HSP90α mRNA was quantified in megakaryocytes and platelets from Sham (*n* = 4) and CLP sepsis mice (*n* = 5). **(D** and **E)** HSP90α mRNA was quantified in platelets from Sham (*n* = 5) and LPS sepsis mice (*n* = 5). **(F** and **G)** HSP90α mRNA was quantified in megakaryocytes and platelets from WT (*n* = 5) and TLR4^-/-^ CLP sepsis mice (*n* = 5). **(H** and **I)** HSP90α mRNA were quantified in megakaryocytes and platelets from WT (*n* = 5) and TLR4^-/-^ LPS sepsis mice (*n* = 5). **(J)** HSP90α mRNA were quantified in WT or TLR4^-/-^ megakaryocytes treated with sham PPP or CLP PPP (*n* = 6). **(K)** Immunoblot and quantification analysis of p-NFκB and NFκB in WT or TLR4^-/-^ megakaryocytes from sham or CLP mice (*n* = 5). All data are presented as the mean ± SD. Statistical analysis was conducted using an unpaired two-tailed *t*-test (B–I) and one-way ANOVA and Holm-Šídák's multiple **
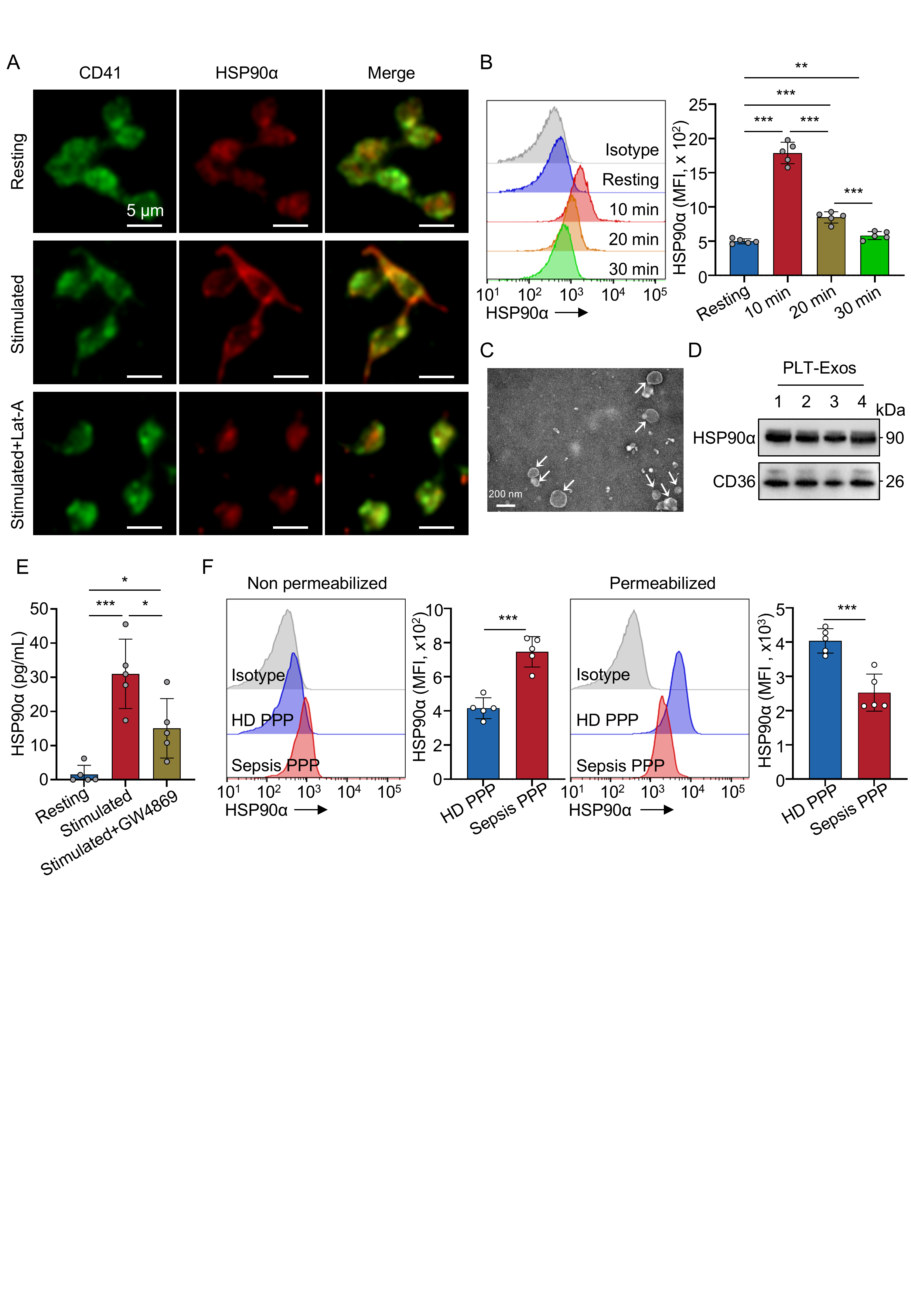
**comparisons test (J, K). ***P* < 0.01, ****P* < 0.001. PPP, platelet-poor plasma.

**Figure S3.** **Platelet HSP90α is translocated and released following thrombin stimulation.** (A) Representative immunofluorescence microscopy of HSP90α (red) and CD41 (green) was performed on sepsis platelets treated for 10 min with HEPES, thrombin (0.05 U/mL), and a combination of latrunculin A (200 μM) and thrombin (0.05 U/mL). Scale bars: 5 μm. (B) Representative flow cytometry histograms and quantification for HSP90α on the membrane surface in resting platelets or stimulated with thrombin (0.05 U/mL) for 10, 20 or 30 minutes (*n* = 5). (C) Electron micrograph of exosomes (indicated by white arrows) derived from the plasma of HD. Scale bar, 200 nm. (D) The expression of HSP90α in thrombin-stimulated platelet-derived exosomes was detected by western blot. (E) ELISA analysis of HSP90α in the supernatant of HD platelets treated with thrombin (0.05 U/mL) or thrombin (0.05 U/mL) combined with GE4869 (20 μM) (*n* = 5). (F) Representative flow cytometry histograms and quantification for HSP90α on the membrane and intracellular in platelets treated with HD (*n* = 5) or sepsis microvesicles/exosomes-depleted PPP (*n* = 5). All data are presented as the mean ± SD. Statistical analysis was conducted using an unpaired two-tailed t-test (F) and one-way ANOVA and Holm-Šídák's multiple comparisons test (B, E). **P < 0.01, ***P < 0.001. PPP,
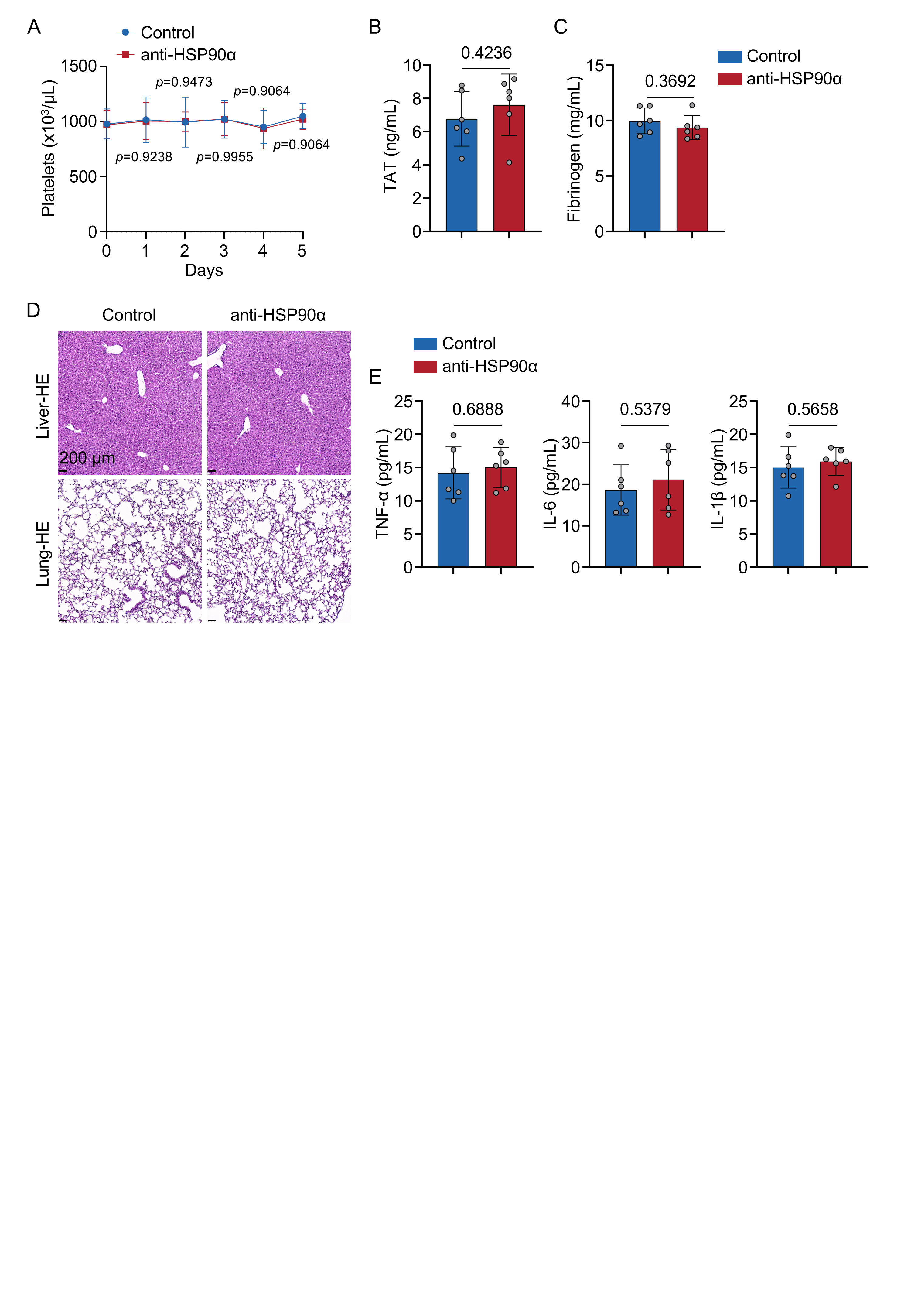
platelet-poor plasma.

**Figure S4. The** **safety assessment of anti-HSP90α antibody treatment in mice. (A)** Platelet counts were assessed using a hematology analyzer during the anti-HSP90α antibody treatment (*n* = 6). **(B)** Plasma TAT level in mice after the anti-HSP90α antibody treatment (*n* = 6). **(C)** Plasma fibrinogen level in mice after the anti-HSP90α antibody (5 mg/kg) treatment (*n* = 6). **(D)** Representative HE staining of liver and lung in mice after the anti-HSP90α antibody treatment (*n* = 6). **(E)** Plasma TNF-α, IL-6 and IL-1β levels in mice after the anti-HSP90α antibody treatment (*n* = 6). All data are presented as the mean ± SD. Statistical analysis was conducted using an unpaired two-tailed *t*-test (A, B, C, E).


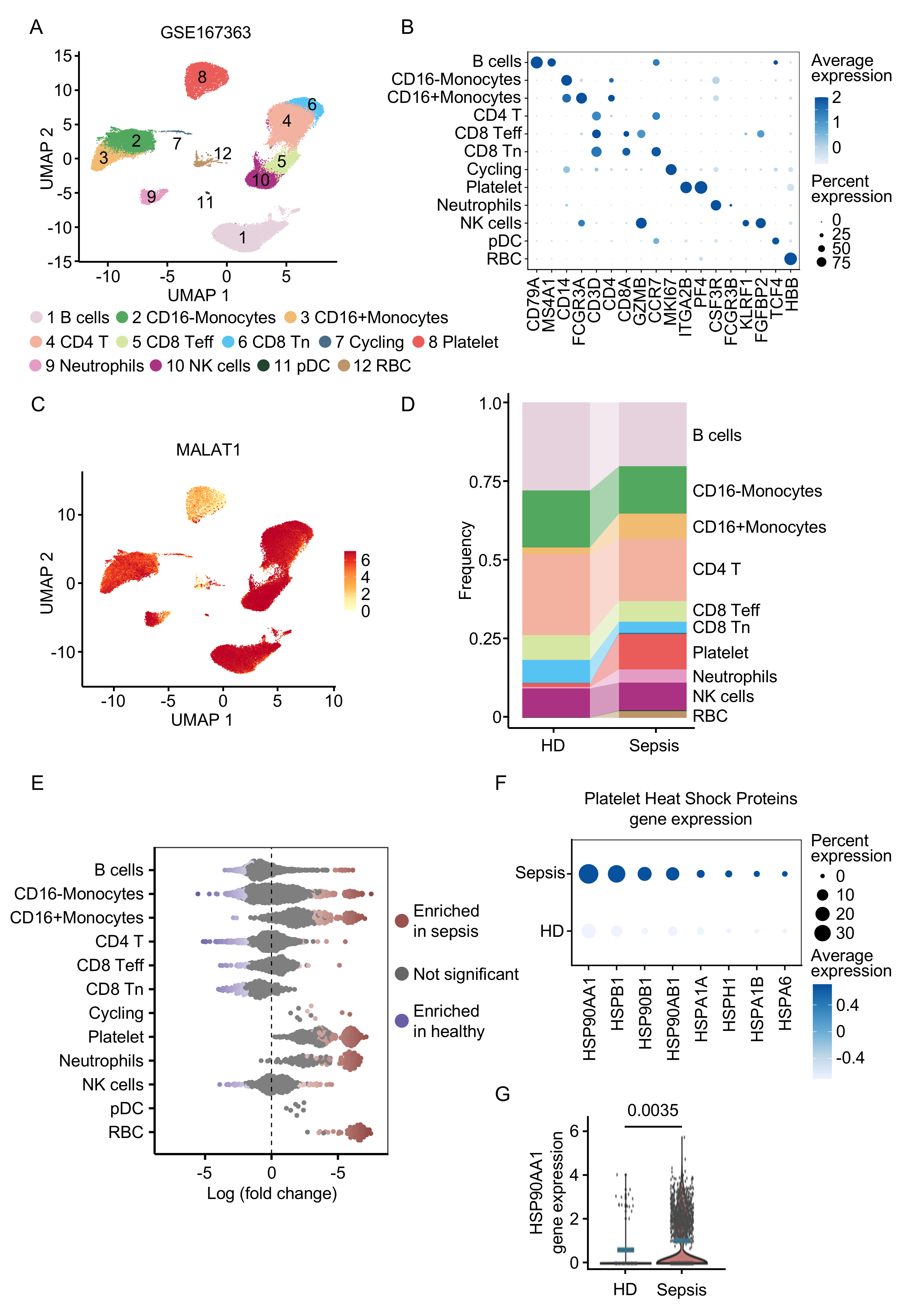
**Figure S5.** **Single cell transcriptomic analysis of PBMC in HD and patients with sepsis.** **(A)** A uniform manifold approximation and projection analysis of human PBMC from HD and patients with sepsis identified 12 distinct clusters. **(B)** Dot plot analysis from scRNA-seq displaying expression of marker genes used to annotate clusters. **(C)** UMAP showing *MALAT1* gene expression within each annotated cluster. **(D)** Graphics showing the relative representation of each annotated cluster from HD and patients with sepsis. **(E)** Violin plot showing the log fold-change of cell type abundance or gene expression across three groups. **(F)** Dot plot analysis from scRNA-seq displaying expression of several HSPs in platelets from HD and sepsis. **(G)** Violin plots with HSP90AA1 expression in platelets of HD and sepsis patients from the scRNA-seq data.

**
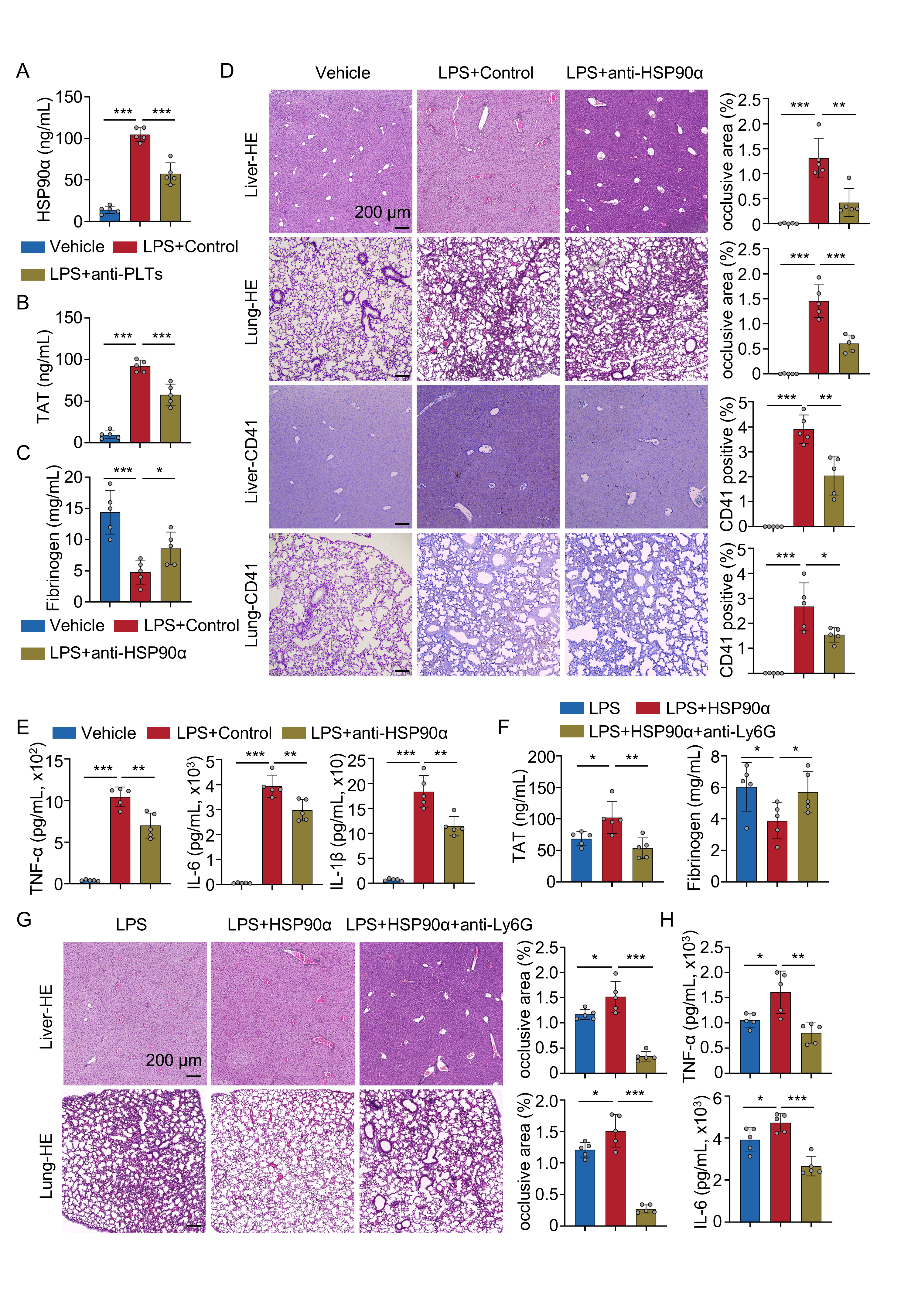
Figure S6.** **eHSP90α facilitated sepsis-induced thrombosis by neutrophils.** **(A)** Plasma HSP90α levels of sham and LPS-treated mice in the absence or presence of anti-PLTs treatment (*n* = 5). **(B)** Plasma TAT levels of sham and LPS-treated mice in the absence or presence of anti-HSP90α treatment (*n* = 5). **(C)** Plasma fibrinogen levels of sham and LPS-treated mice in the absence or presence of anti-HSP90α treatment (*n* = 5). **(D)** HE staining and IHC staining of CD41 in livers and lungs of sham and LPS-treated mice in the absence or presence of anti-HSP90α treatment (*n* = 5). HE staining statistical analysis for thrombus area in mice livers and lungs was shown (*n* = 5). IHC staining statistical analysis for platelets in mice livers and lungs is shown (*n* = 5). Scale bars: 200 μm. **(E)** Bar graphs displaying the levels of TNF-α, IL-6, and IL-1β in plasma from each group. **(F)** Plasma TAT and fibrinogen levels of LPS-treated mice, LPS-treated mice treated with recombinant HSP90α and/or anti-Ly6G antibody. (*n* = 5). **(G)** HE staining in livers and lungs of LPS-treated mice, LPS-treated mice treated with recombinant HSP90α and/or anti-Ly6G antibody (*n* = 5). HE staining statistical analysis for thrombus area in mice livers and lungs was shown (*n* = 5). **(H)** Bar graphs displaying the levels of TNF-α and IL-6 in plasma from each group. All data are presented as the mean ± SD. Statistical analysis was conducted using one-way ANOVA and Holm-Šídák's multiple comparisons test (A–H). **P* < 0.05, ***P* < 0.01, ****P* < 0.001.

**
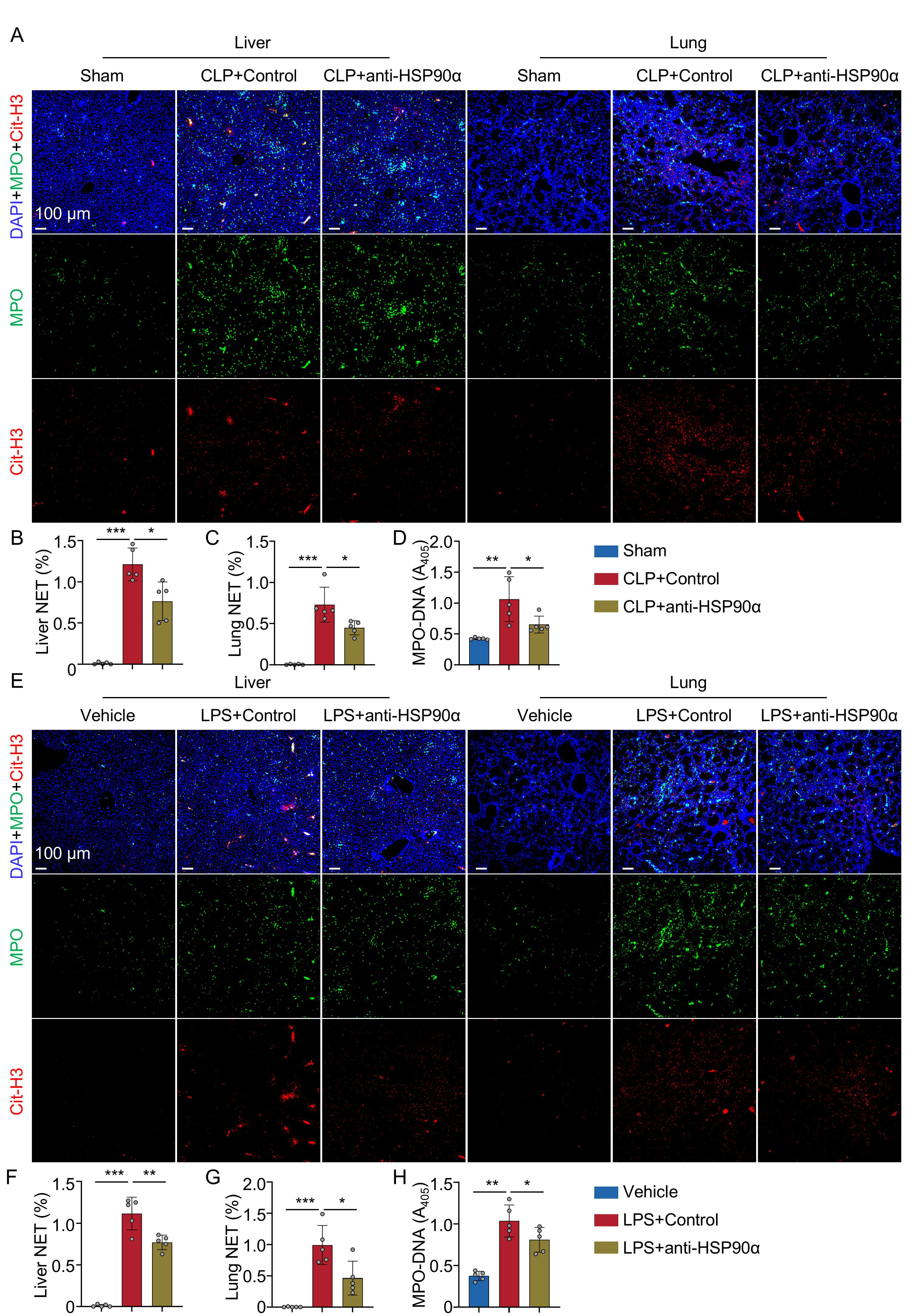
Figure S7.** **1G6-D7 administration substantially inhibited the NETs formation in both CLP and LPS mice.** (**A**–**C)** Representative confocal microscopy and quantification of NETs in liver and lung of sham and CLP-operated mice (*n* = 5) in absence or presence of anti-HSP90α treatment (*n* = 5). Cells were stained with DAPI for DNA (blue), anti-MPO (green) and anti-Cit-H3 (red) for NETs. Scale bars: 100 μm. **(D)** Quantification of MPO-DNA in the plasma from each group (*n* = 5). (**E**–**G)** Representative confocal microscopy and quantification of NETs in liver and lung of sham and LPS-treated mice (*n* = 5) in absence or presence of anti-HSP90α treatment (*n* = 5). Cells were stained with DAPI for DNA (blue), anti-MPO (green) and anti-Cit-H3 (red) for NETs. Scale bars: 100 μm. **(H)** Quantification of MPO-DNA in the plasma from each group (*n* = 5). All data are presented as the mean ± SD. Statistical analysis was conducted using one-way ANOVA and Holm-Šídák's multiple comparisons test (A–H). **P* < 0.05, ***P* < 0.01, ****P* < 0.001.


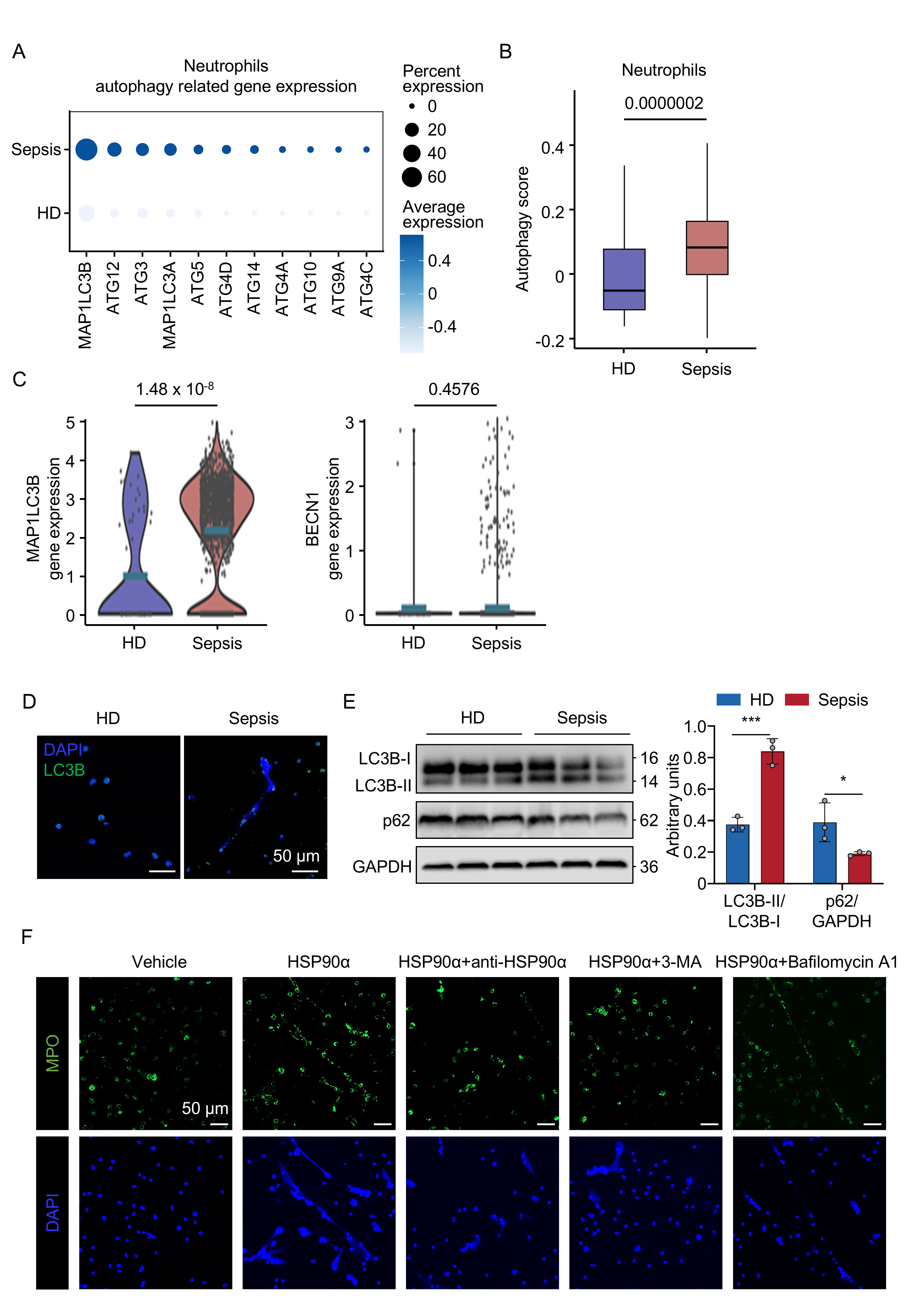
**Figure S8.** **Elevated autophagy in neutrophils from patients with sepsis.** **(A)** Dot plot analysis from scRNA-seq (GSE167363) displaying autophagy-related gene expression in neutrophils from HD and sepsis. **(B)** Bar graphics showing the autophagy score in neutrophils from HD and sepsis. (**C** and **D)** Violin plots with MAP1LC3B and BECN1 expression in neutrophils of HD and sepsis patients from the scRNA-seq data. (**E)** Immunofluorescence analysis of LC3B (green) and DAPI (blue) was performed in neutrophils from HD and sepsis patients. Scale bars: 50 μm. **(F)** Immunoblot and quantification analysis for LC3B and p62 in neutrophils from HD (*n* = 3) and sepsis patients (*n* = 3). **(G)** The individual green (MPO) and blue (DAPI) channels related to Figure 5E. All data are presented as the mean ± SD. Statistical analysis was conducted using an unpaired two-tailed *t*-test (E). **P* < 0.05. LC3B, microtubule associated protein 1 light chain 3 beta.

**
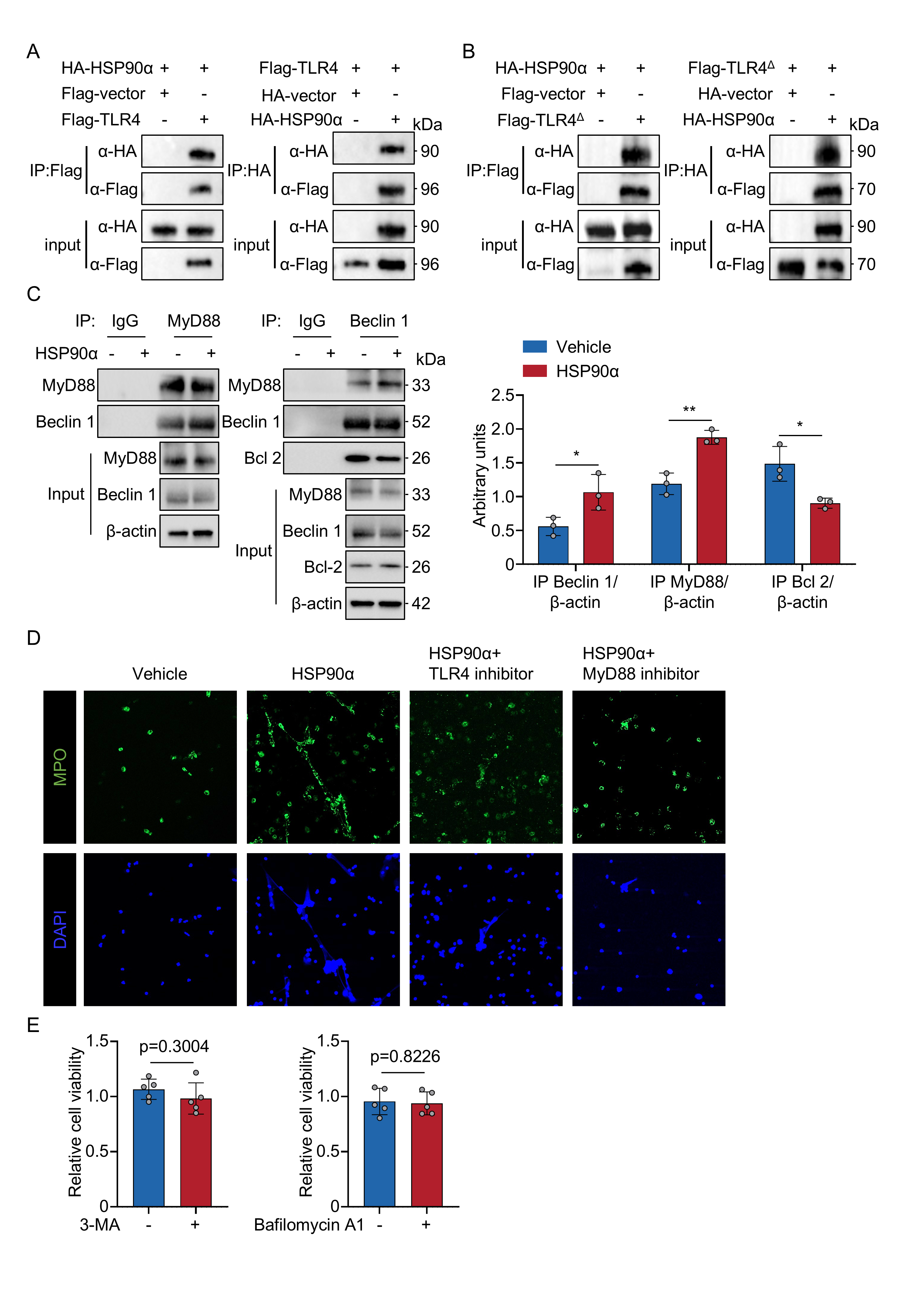
Figure S9.** **eHSP90α exerts its function through the TLR4-MyD88 signaling axis. (A)** The immunoprecipitation with anti-Flag and anti-HA antibody was performed using 293T cells transfected with Flag-TLR4 in combination with HA-HSP90α or alone. **(B)** The immunoprecipitation with anti-Flag and anti-HA antibody was performed using 293T cells transfected with Flag-TLR4 extracellular domain (24-631 aa) (Flag-TLR4^Δ^) in combination with HA-HSP90α or alone. **(C)** The quantification analysis of immunoprecipitation with anti-MyD88 and anti-Beclin 1 antibody in neutrophils incubated with or without 10 μg/mL HSP90α (*n* = 3) (related to Figure 6D). **(D)** The individual green (MPO) and blue (DAPI) channels related to Figure 6F. **(E)** Cell viability was determined by CCK8 assay in neutrophils incubated with 3-MA (5 mM) (*n* = 5) or bafilomycin A1 (1μM) (*n* = 5). All data are presented as the mean ± **
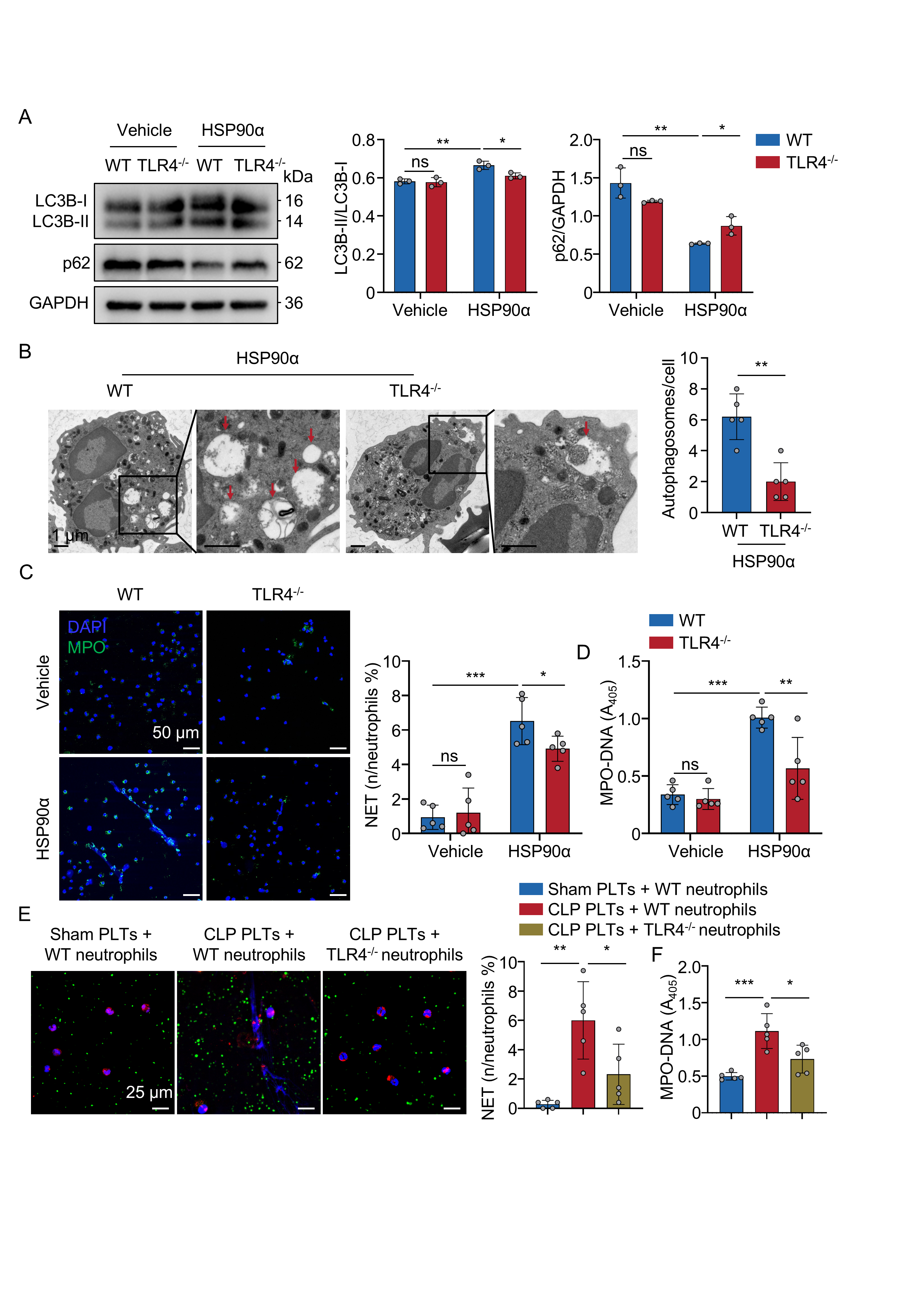
**SD. Statistical analysis was conducted using an unpaired two-tailed *t*-test (C, E). **P* < 0.05.

**Figure S10. eHSP90α functions through its receptor TLR4.** **(A)** Immunoblot and quantification analysis for LC3B and p62 in WT (*n* = 3) or TLR4^-/-^ (*n* = 3) neutrophils incubated with recombinant HSP90α (10 μg/mL). **(B)** Representative transmission electron microscope of WT and TLR4^-/-^ neutrophils stimulated with recombinant HSP90α (10 μg/mL). Scale bar: 1 μm. Red arrowheads denote autolysosomes. Autophagic vesicles (autophagosome/autolysosome) were quantified per cell. **(C)** Immunofluorescence and quantification analysis of NETs in WT or TLR4^-/-^ neutrophils incubated with recombinant HSP90α (10 μg/mL) (*n* = 5). Scale bars: 50 μm. **(D)** Quantification of MPO-DNA in the supernatant of neutrophils from each group (*n* = 5). **(E)** Immunofluorescence and quantification analysis of NETs following in vitro co-culture of WT or TLR4^-/-^ neutrophils with Sham or CLP PLTs (*n* = 5). Scale bars: 25 μm. **(F)** Quantification of MPO-DNA in the supernatant of neutrophils from each group (*n* = 5). All data are presented as the mean ± SD. Statistical analysis was conducted using one-way ANOVA and Holm-Šídák's multiple comparisons test (A, C, D, E, F) and unpaired two-tailed *t*-test (B). **P* < 0.05, ***P* < 0.01, ****P* < 0.001.

**Table 1** Characteristics of the study population

| Characteristics | HD (n=20) | Sepsis (n=20) |
| --- | --- | --- |
| Age (years) | 4.70±3.49 | 3.70±3.40 |
| Male (%) | 65% | 80% |
| WBC (10^9^/L) | 8.48±2.35 | 11.92±10.37 |
| PMNs (10^9^/L) | 4.40±2.25 | 7.44±8.10 |
| Lymphocyte (10^9^/L) | 3.48±1.73 | 3.69±4.75 |
| Monocyte (10^9^/L) | 0.39±0.22 | 0.66±0.60 |
| Eosinophil (10^9^/L) | 0.18±0.23 | 0.09±0.16 |
| Basophil (10^9^/L) | 0.03±0.02 | 0.03±0.03 |
| N (%) | 51.13±20.29 | 58.58±24.32 |
| L (%) | 42.09±18.40 | 34.50±22.86 |
| RBC (10^12^/L) | 4.49±0.55 | 3.98±0.74* |
| HGB (g/L) | 120.85±11.89 | 108.1±22.02* |
| PLT (10^9^/L) | 342.40±105.20 | 293.90±133.30 |
| MPV (fL) | 9.47±0.75 | 10.07±1.51 |
| PDW (fL) | 9.98±1.48 | 11.31±3.37 |
| P-LCR (%) | 20.27±6.31 | 24.61±11.33 |
| CRP (mg/L) | 2.55±3.66 | 36.15±47.33** |
| PCT (ng/L) | 0.13±0.10 | 2.57±4.65* |
| APTT (S) | 31.16±3.77 | 31.89±5.25* |
| PT (S) | 12.03±0.84 | 12.97±1.39* |
| FIB (g/L) | 2.74±0.79 | 8.43±16.17 |
| ALT (U/L) | 21.54±13.27 | 61.09±70.70* |
| AST (U/L) | 38.51±14.79 | 59.47±59.48 |
| LDH (U/L) | 286.69±101.30 | 326.01±113.84 |
| ALB (g/L) | 41.67±4.31 | 37.81±4.74* |
| Septic shock | n = 0 | n = 3 |

Data are presented as median ± SD. Statistical analysis was conducted using unpaired two-tailed *t*-test. *P < 0.05, **P < 0.01. WBC, white blood cell; PMNs, polymorphonuclear neutrophils; N %, PMN to white blood cell ratio; L %, lymphocyte to white blood cell ratio; RBC, red blood cell; HGB, hemoglobin; PLT, platelets; MPV, mean platelet volume, PDW, platelet distribution width; P-LCR, platelet large cell ratio; CRP, C-reactive protein; PCT, procalcitonin; APTT, activated partial thromboplastin time; PT, prothrombin time; FIB, fibrinogen; ALT, alanine transaminase; AST; aspartate aminotransferase; LDH, lactic dehydrogenase; ALB, albumin.
